# A common form of dominant human IFNAR1 deficiency impairs IFN-α and -ω but not IFN-β-dependent immunity

**DOI:** 10.1084/jem.20241413

**Published:** 2024-12-16

**Authors:** Fahd Al Qureshah, Jérémie Le Pen, Nicole A. de Weerd, Marcela Moncada-Velez, Marie Materna, Daniel C. Lin, Baptiste Milisavljevic, Fernanda Vianna, Lucy Bizien, Lazaro Lorenzo, Marc Lecuit, Jean-David Pommier, Sevgi Keles, Tayfun Ozcelik, Sigifredo Pedraza-Sanchez, Nicolas de Prost, Loubna El Zein, Hassan Hammoud, Lisa F.P. Ng, Rabih Halwani, Narjes Saheb Sharif-Askari, Yu Lung Lau, Anthony R. Tam, Neha Singh, Sagar Bhattad, Yackov Berkun, Wasun Chantratita, Raúl Aguilar-López, Mohammad Shahrooei, COVID-19 HGE Consortium, Laurent Abel, Alessandro Aiuti, Saleh Al-Muhsen, Ana Bertha Alcántara-Garduño, Evangelos Andreakos, Andrés A. Arias, Hagit Baris Feldman, Paul Bastard, Alexandre Bolze, Alessandro Borghesi, Ahmed A. Bousfiha, Petter Brodin, John Christodoulou, Aurélie Cobat, Roger Colobran, Antonio Condino-Neto, Sotiriјa Duvlis, Xavier Duval, Munis Dündar, Soha Fakhreddine, Jacques Fellay, Carlos Flores, José Luis Franco, Guy Gorochov, Peter K. Gregersen, David Hagin, Rabih Halwani, María Teresa Herrera, Ivan Fan-Ngai Hung, Emmanuelle Jouanguy, Yu-Lung Lau, Daniel Leung, Tom Le-voyer, Davood Mansouri, Jesús Mercado-García, Isabelle Meyts, Trine H. Mogensen, Lisa F.P. Ng, Antonio Novelli, Giuseppe Novelli, Satoshi Okada, Firat Ozcelik, Tayfun Ozcelik, Rebeca Perez de Diego, Jordi Perez-Tur, Graziano Pesole, Anne Puel, Laurent Renia, Igor Resnick, Carlos Rodríguez-Gallego, Manal Sbeity, Sahar Sedighzadeh, Mohammad Shahrooei, Pere Soler-Palacín, András N. Spaan, Stuart G. Tangye, Ahmad Abou Tayoun, Şehime Gülsün Temel, Christian Thorball, Ibrahim Torktaz, Sophie Trouillet-Assant, Stuart E. Turvey, Furkan Uddin, Fernanda Sales Luiz Vianna, Donald C. Vinh, Oscar Zabaleta-Martínez, Qian Zhang, Shen-Ying Zhang, Jean-Laurent Casanova, SEAe Consortium, Chanreaksmey Eng, Kimrong Bun, MengHeng Oum, Patrice Piola, Arnaud Tarantola, Mey Channa, Veasna Duong, Philippe Buchy, Chris Gorman, Jean-David Pommier, Yoann Crabol, Philippe Dussart, M. Bunleat, M. Panha, M.Kanarith Sim, Em Bunnakea, Denis Laurent, Heng Sothy, Ky Santy, Anousone Douangnouvong, Danoy Chommanam, Khansoudaphone Phakhounthong, Manivanh Vongsouvath, Malee Seephone, Bountoy Sibounheunang, Sayaphet Rattanavong, Viengmon Davong, Malavanh Vongsouvath, Mayfong Mayxay, Audrey Dubot-Pérès, Paul N. Newton, Sommanikhone Phangmanixay, Khounthavy Phongsavath, Dang Duc Anh, Do Quyen, Tran Thi Mai Hung, Nguyen Thi Thu Thuy, Luong Minh Tan, Anh Tuan Pham, Nguyen Hien, Do Thu Huong, Le Thanh Hai, Nguyen Van Lam, Pham Nhat An, Phan Huu Phuc, Phung Bich Thuy, Tran Thi Thu Huong, Chaw Su Hlaing, Aye Mya Min Aye, Cho Thair, Kyaw Linn, May July, Win Thein, Latt Latt Kyaw, Htay Htay Tin, Ommar Swe Tin, Khin Yi Oo, Yoann Crabol, Magali Herrant, Magali Lago, Maud Seguy, Marc Jouan, Lukas Hafner, Philippe Pérot, Marc Eloit, Marc Lecuit, Olivier Lortholary, Julien Capelle, Bruno Rosset, Veronique Chevalier, Jérôme Honnorat, Anne Laurie Pinto, Auey Dubot-Peres, Xavier de Lamballerie, Kevin Bleakley, Bernadette Murgue, Catherine Ferrant, Christian Devaux, Hervé Tissot-Dupont, Jean-Paul Moatti, Mayfong Mayxay, Pascal Bonnet, Didier Fontenille, Jean-François Delfraissy, Patrice Debré, Benoit Durand, Laurent Abel, Paul Bastard, Emmanuelle Jouanguy, Vivien Béziat, Peng Zhang, Charles M. Rice, Aurélie Cobat, Shen-Ying Zhang, Paul J. Hertzog, Jean-Laurent Casanova, Qian Zhang

**Affiliations:** 1 St Giles Laboratory of Human Genetics of Infectious Diseases, Rockefeller Branch, Rockefeller University, New York, NY, USA; 2 Wellness and Preventive Medicine Institute, King Abdulaziz City for Science and Technology, Riyadh, Saudi Arabia; 3 Laboratory of Virology and Infectious Disease, The Rockefeller University, New York, NY, USA; 4 Centre for Innate Immunity and Infectious Diseases, Department of Molecular and Translational Science, Hudson Institute of Medical Research and Monash University, Clayton, Australia; 5 Laboratory of Human Genetics of Infectious Diseases, INSERM U1163, Necker Hospital for Sick Children, Paris, France; 6 Université Paris Cité, Imagine Institute, Paris, France; 7 Laboratório de Medicina Genômica Centro de Pesquisa Experimental, Hospital de Clínicas de Porto Alegre, Porto Alegre, Brazil; 8 Graduate Program in Genetics and Molecular Biology, Federal University of Rio Grande do Sul, Porto Alegre, Brazil; 9 Graduate Program in Medicine, Medical Sciences, Federal University of Rio Grande do Sul, Porto Alegre, Brazil; 10 National Institute of Population Medical Genetics (INAGEMP), Porto Alegre, Brazil; 11 Department of Infectious Diseases and Tropical Medicine, Necker-Enfants Malades University Hospital, APHP, Institut Imagine, Paris, France; 12 Biology of Infection Unit, Institut Pasteur, Inserm U1117, Université Paris Cité, Paris, France; 13 Division of Pediatric Allergy and Immunology, Meram Medical Faculty, Necmettin Erbakan University, Konya, Turkey; 14 Department of Molecular Biology and Genetics, Bilkent University, Bilkent-Ankara, Turkey; 15 Unit of Biochemistry, National Institute for Medical Sciences and Nutrition Salvador Zubiran (INCMNSZ), Mexico City, Mexico; 16 Service de Médecine Intensive Réanimation, Hôpitaux Universitaires Henri Mondor, Assistance Publique-Hôpitaux de Paris (AP-HP), Paris, France; 17 Groupe de Recherche Clinique CARMAS, Faculté de Santé de Créteil, Université Paris Est Créteil, Créteil Cedex, France; 18 INSERM U955, Team “Viruses, Hepatology, Cancer”, Créteil, France; 19 Biology Department, Lebanese University, Beirut, Lebanon; 20 Saint Georges Hospital, Beirut, Lebanon; 21 A*STAR Infectious Disease Labs, Agency for Science, Technology and Research, Singapore, Singapore; 22 Lee Kong Chian School of Medicine, Nanyang Technology University, Singapore, Singapore; 23 Research Institute for Medical and Health Sciences, University of Sharjah, Sharjah, UAE; 24 Prince Abdullah Bin Khalid Celiac Disease Research Chair, Department of Pediatrics, Faculty of Medicine, King Saud University, Riyadh, Saudi Arabia; 25 Department of Pediatrics and Adolescent Medicine, The University of Hong Kong, Hong Kong, China; 26 Division of Infectious Diseases, Department of Medicine, School of Clinical Medicine, University of Hong Kong, Hong Kong, China; 27 ASTER CMI Hospitals, Bengaluru, India; 28 Department of Pediatrics, Hadassah-Hebrew University Medical Center, Mount Scopus and Faculty of Medicine, Hebrew University of Jerusalem, Jerusalem, Israel; 29 Center for Medical Genomics, Faculty of Medicine Ramathibodi Hospital, Mahidol University, Bangkok, Thailand; 30 Department of Surgery, Maternal and Child Hospital, Social Security Institute of the State of Mexico and Municipalities (ISSEMYM), Toluca, Mexico; 31 Clinical and Diagnostic Immunology, Department of Microbiology, Immunology, and Transplantation, KU Leuven, Leuven, Belgium; 32 Dr. Shahrooei’s Laboratory, Tehran, Iran; 33 Pediatric Hematology-Immunology and Rheumatology Unit, Necker Hospital for Sick Children, Assistance Publique-Hôpitaux de Paris, Paris, France; 34 Howard Hughes Medical Institute, New York, NY, USA; 35 Department of Pediatrics, Necker Hospital for Sick Children, Paris, France

## Abstract

Autosomal recessive deficiency of the IFNAR1 or IFNAR2 chain of the human type I IFN receptor abolishes cellular responses to IFN-α, -β, and -ω, underlies severe viral diseases, and is globally very rare, except for IFNAR1 and IFNAR2 deficiency in Western Polynesia and the Arctic, respectively. We report 11 human *IFNAR1* alleles, the products of which impair but do not abolish responses to IFN-α and -ω without affecting responses to IFN-β. Ten of these alleles are rare in all populations studied, but the remaining allele (P335del) is common in Southern China (minor allele frequency ≈2%). Cells heterozygous for these variants display a dominant phenotype in vitro with impaired responses to IFN-α and -ω, but not -β, and viral susceptibility. Negative dominance, rather than haploinsufficiency, accounts for this dominance. Patients heterozygous for these variants are prone to viral diseases, attesting to both the dominance of these variants clinically and the importance of IFN-α and -ω for protective immunity against some viruses.

## Teaser

This study identifies 11 IFNAR1 alleles impairing IFN-α/ω responses but sparing IFN-β, including an allele common in Southern China (P335del). Heterozygous variants show dominant negative effects, linking these variants to increased susceptibility to respiratory and cerebral viral diseases.

## Introduction

Human type I interferons (IFNs) form a family of 16 subtypes encoded by 17 intron-less genes: 12 IFN-α subtypes (13 loci, 2 encoding the same protein), IFN-β, IFN-κ, IFN-ω, and IFN-ɛ, all of which bind to the type I IFN receptor composed of the IFNAR1 and IFNAR2 chains (Wittling et al., 2021). IFN-β is the IRF7-independent, high-affinity, short-lived, autocrine IFN that initiates the induction of the other IFNs (Randall and Goodbourn, 2008; Wittling et al., 2021). IFN-κ and IFN-ɛ have low affinity and are expressed in the skin and uterus, respectively (Wittling et al., 2021). The single IFN-ω and the 12 IFN-α are produced in abundance by leukocytes, including plasmacytoid dendritic cells, and circulate within body fluids. The affinity of IFN-ω for its receptor is lower than that of IFN-β but higher than that of all IFN-α (Wittling et al., 2021). The cellular detection of viral infection triggers the production of type I IFNs. Most, if not all cells can make and respond to at least one type I IFN. The binding of type I IFNs to their receptor leads to the activation of JAK1 and TYK2, which in turn phosphorylates and activates STAT1 and STAT2 (Duncan et al., 2021). The phosphorylated STAT1 and STAT2 proteins form a heterodimer that binds to IRF9 to form the interferon-stimulated gene factor protein complex (ISGF3). The ISGF3 complex is translocated to the nucleus, where it binds to IFN-stimulated response elements (ISREs) to mediate the induction of IFN-stimulated genes (ISGs), which have various effects, some of which are antiviral (Park and Iwasaki, 2020; Diamond and Kanneganti, 2022; Schneider et al., 2014). Type I IFN activity requires precise regulation because excessive activity can lead to type I interferonopathies (Crow and Stetson, 2022), whereas genetic deficiencies of type I IFN immunity underlie various viral illnesses (Meyts and Casanova, 2021; Duncan et al., 2021; Crow and Casanova, 2024).

The critical role of human type I IFNs in protective immunity against viral infections was demonstrated by the discovery of patients with monogenic inborn errors of immunity (IEIs) of genes encoding either of the two chains of the receptor for type I IFNs. These patients are at high risk of developing life-threatening viral infections. Autosomal recessive (AR) complete IFNAR1 deficiency has been shown to underlie adverse reactions to vaccination with live-attenuated viruses (LAV), including measles–mumps–rubella (MMR) and yellow fever (YF) (Hernandez et al., 2019; Bastard et al., 2022a; Gothe et al., 2022). Herpes simplex virus 1 (HSV-1) encephalitis (HSE) has been reported in a patient with a distinctive form of AR complete IFNAR1 deficiency in which a non-functional IFNAR1 is expressed on the cell surface at levels similar to those typically observed for the wildtype IFNAR1 (Bastard et al., 2021b). AR IFNAR1 deficiency has recently been detected in previously healthy unrelated adults with hypoxemic COVID-19 pneumonia (Zhang et al., 2020), and AR IFNAR1 deficiency has been reported in four children with hypoxemic COVID-19 pneumonia (Khanmohammadi et al., 2022; Zhang et al., 2022b; Abolhassani et al., 2022). Patients with IFNAR2 deficiencies have also been reported to develop adverse reactions to MMR or YF vaccines (Duncan et al., 2015, 2022; Bastard et al., 2021c; Passarelli et al., 2020) and life-threatening COVID-19 or influenza (Zhang et al., 2020; Duncan et al., 2022). Collectively, these findings highlight the importance of type I IFNs for immunity to both LAV and naturally acquired respiratory and cerebral viruses. Surprisingly, the patients concerned had managed to live to the age of 1–38 years without experiencing other unusually severe viral illnesses, and some are still alive at the age of 45–55 years. Surprisingly, loss-of-function alleles of *IFNAR1* and *IFNAR2* were found to be common in Western Polynesians and Arctic peoples with an MAF >1% (1.25% and 3.4%, respectively) (Bastard et al., 2022a; Duncan et al., 2022), despite their absence or extreme rarity elsewhere. The frequency of homozygotes in these isolated populations has been estimated at 1/6,450 in Samoa and 1/1,539 in Greenland, and these individuals appear to be prone to only a few severe viral diseases.

The essential role of human type I IFNs in protective immunity to certain viruses was clearly illustrated by the discovery of autoantibodies (auto-Abs) neutralizing type I IFNs, especially IFN-α and/or -ω, and rarely IFN-β (Bastard et al., 2022b, 2024; Hale, 2023). These auto-Abs block the protective antiviral function of IFNs and underlie ∼15% of critical COVID-19 pneumonia cases (Bastard et al., 2020, 2021a; Manry et al., 2022), ∼30% of severe infections following vaccination against YF (Bastard et al., 2021c), ∼5% of severe influenza pneumonia cases (Zhang et al., 2022c), ∼25% of hospitalizations for Middle East respiratory syndrome (MERS) pneumonia (Alotaibi et al., 2023), and severe cases of herpetic infections (Pozzetto et al., 1984; Hetemäki et al., 2021; Busnadiego et al., 2022). Auto-Abs neutralizing IFN-α and/or IFN-ω have also recently been shown to underlie ∼40% of cases of West Nile virus encephalitis, the most severe form of West Nile disease (Gervais et al., 2023), and ∼10% of cases of severe tick-borne encephalitis (TBE) (Gervais et al., 2024). Patients with neutralizing auto-Abs against type I IFN can, thus, be considered to display autoimmune, partial phenocopies of AR IFNAR1 or IFNAR2 deficiency (Casanova et al., 2024). Some IEIs, such as AIRE deficiencies in *cis* and in *trans*, underlie the production of autoantibodies against type I IFNs (Meager et al., 2006; Le Voyer et al., 2023). Viral diseases can, therefore, be due to IEIs that directly disrupt the type I IFN signaling pathway or by IEIs that disrupt tolerance to type I IFNs (Su et al., 2023; Casanova and Anderson, 2023). Cellular responses to type I IFNs in these patients depend on the levels and affinities of the auto-Abs present and the specific subtypes of IFN neutralized (Manry et al., 2022). In this context, we tested the hypothesis that there might be new genetic forms of inherited IFNAR1 deficiency in patients with life-threatening viral diseases. We systematically analyzed all monoallelic and biallelic, and rare and common IFNAR1 variants in our cohort of patients with viral diseases.

## Results

### *IFNAR1* variants in our database and in gnomAD

**Figure S1.**
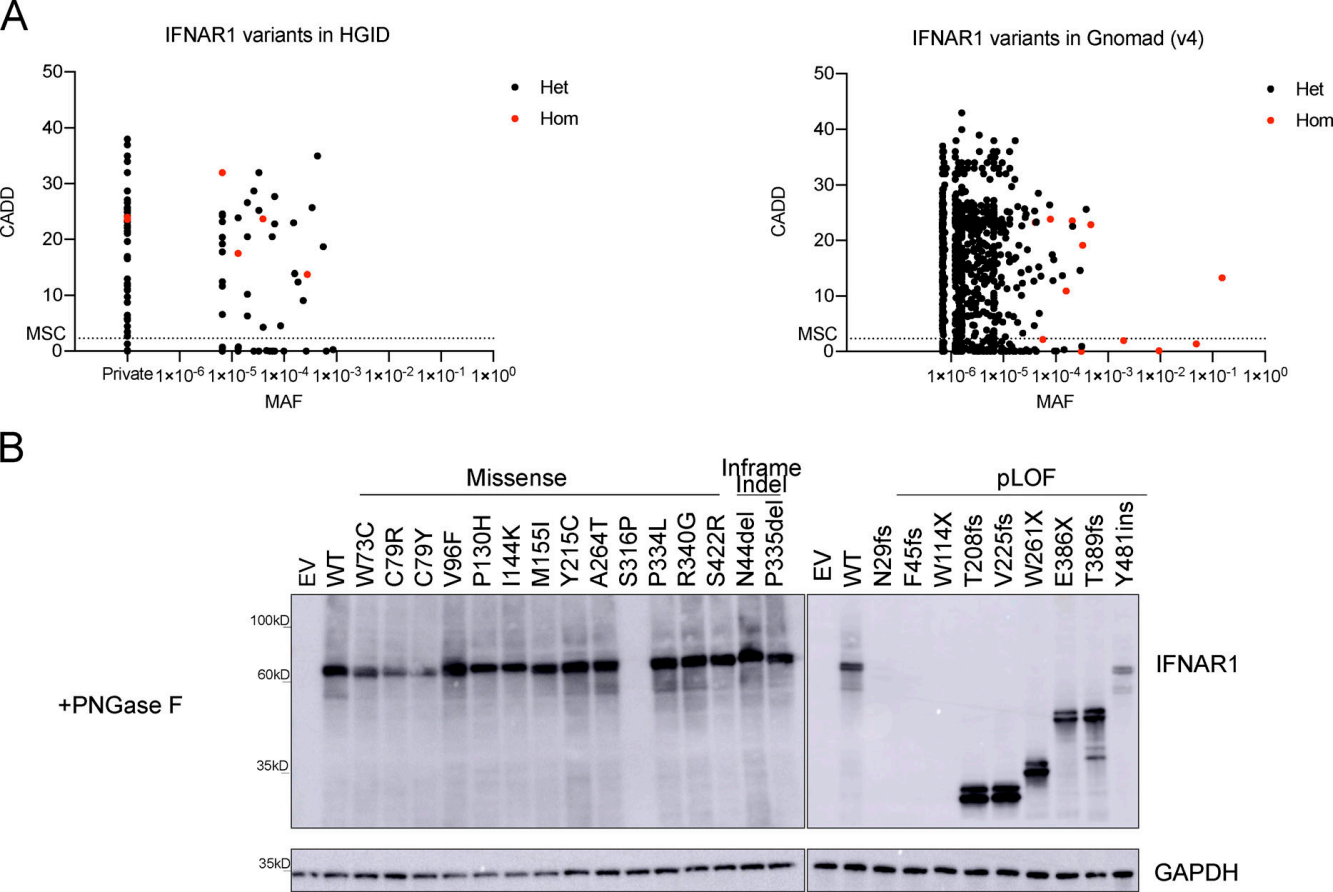
**Population genetics of the *IFNAR1* variants present in the HGID and gnomAD v4.0.0 databases. (A)** The biallelic variants are shown in red, whereas the monoallelic variants are shown in black. The dotted line represents the gene damage index. MSC, mutation significance cutoff; CADD, combined annotation-dependent depletion; MAF, minor allele frequency. **(B)** Western blot for IFNAR1 in IFNAR1-deficient HEK293T cells transiently transfected with WT or mutant IFNAR1 cDNA constructs and treated with PNGase to remove oligosaccharides from glycoproteins. An antibody recognizing the N-terminus (SD2) of the IFNAR1 protein was used. GAPDH was used as a loading control. A representative blot from at least two experiments is shown. EV, empty vector; WT, wild type. Source data are available for this figure: SourceData FS1.

We first searched for monoallelic and biallelic non-synonymous *IFNAR1* variants in our Human Genetics of Infectious Diseases (HGID) cohort of 19,489 individuals with various viral diseases, including, but not restricted to HSE, critical COVID-19 and influenza pneumonia, and adverse reactions to LAV (https://www.hgid.org). We searched for missense, in-frame indel, predicted loss-of-function (pLOF), and intronic variants between branchpoints and canonical splice acceptor sites with our new tools BPHunter and AGAIN (Zhang et al., 2022a, 2023). We found 98 monoallelic or biallelic variants of *IFNAR1*, all of which were private or rare (i.e., with a global minor allele frequency [MAF] < 0.01); none were common worldwide, defined as MAF >0.01 (Fig. S1 A). We also investigated a relevant subset of *IFNAR1* variants found in the Genome Aggregation Database (gnomAD; v4.0.0) for the general population. We selected variants that had an MAF >1 × 10^−4^ in gnomAD and/or were reported to be present in the homozygous state in at least one individual (Fig. S1 A). We identified 18 variants, 11 of which were common to our HGID cohort; none of these variants was common worldwide. A total of 105 variants from HGID and/or gnomAD were selected for experimental studies: 91 missense, 4 inframe indels, 1 large deletion, and 9 pLOF variants (3 nonsense, 4 frameshift, and 2 essential splice-site variants). We previously showed that the large deletion and the two splice-site variants disrupt the splicing of *IFNAR1* mRNA and generate abnormal transcripts and truncated proteins (Bastard et al., 2021b). We systematically tested all the *IFNAR1* variants, including those previously reported.

### Functional characterization of *IFNAR1* variants

**Figure 1.**
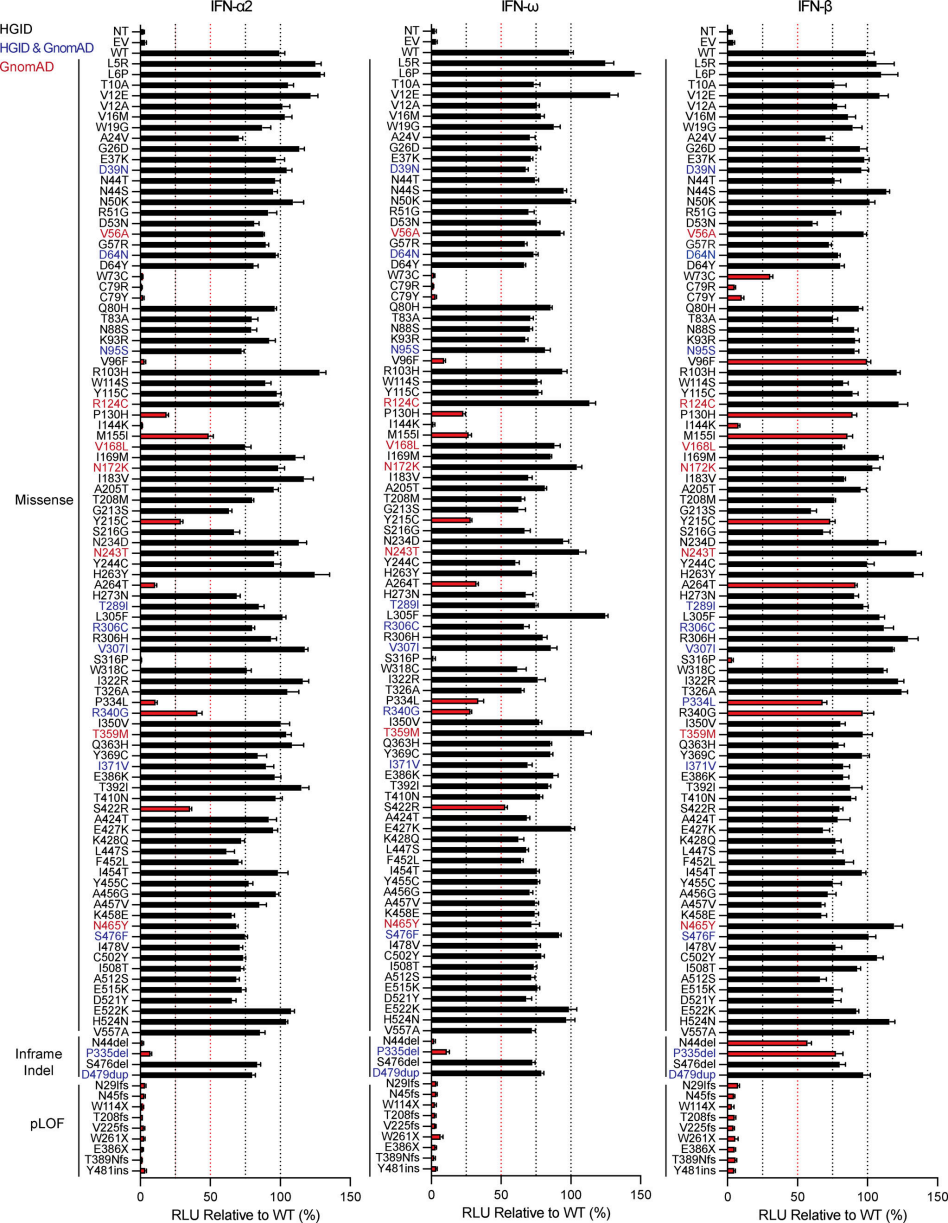
**Functional characterization of IFNAR1 variants.** Luciferase activity in IFNAR1^−/−^ HEK293T cells transiently transfected with WT or mutant IFNAR1 cDNA constructs, together with an ISRE firefly luciferase reporter and a constitutively expressed *Renilla* luciferase reporter, stimulated with IFN-α2 (1,000 U/ml), IFN-ω (1 ng/ml), or IFN-β (100 U/ml) for 24 h. The specific response to IFN stimulation was calculated by determining the ratio of firefly luciferase activity to *Renilla* luciferase activity (RLU, relative luciferase ratio). Variants found only in the HGID cohort are indicated in black, variants unique to gnomAD are indicated in red, and variants common to both are indicated in blue. Hypomorphic or LOF variants are indicated by a red bar. The red line shows the 50% cutoff. NT, non-transfected; EV, empty vector. Graphs depict the mean ± SEM of two independent experiments.

We first screened the *IFNAR1* variants to determine whether the mutations affected the ability of the encoded proteins to respond to IFN-α, IFN-ω, and IFN-β. We used a luciferase reporter assay including ISREs (Bastard et al., 2022a). IFNAR1-deficient HEK293T cells were cotransfected with plasmids encoding the various *IFNAR1* variants and a luciferase reporter plasmid containing five ISREs. They were then stimulated with IFN-α2 (non-glycosylated), IFN-ω (glycosylated), or IFN-β (glycosylated). Variants were classified as deleterious if their luciferase activity was at least two standard deviations below the mean, corresponding to <50% of wild-type activity. LOF and hypomorphic variants were deleterious and had no activity and only residual levels of activity, respectively. We identified 24 deleterious variants: 13 missense, 2 in-frame indel, and 9 pLOF. In HEK293T cells transfected with the nine pLOF variants, luciferase activity in response to IFN-α, IFN-ω, or IFN-β was completely abolished (Fig. 1). Moreover, two in-frame variants (C79R and S316P) were LOF for responses to IFN-α, IFN-ω, and IFN-β (Fig. 1). Interestingly, three in-frame variants (W73C, C79Y, and I144K) were equally hypomorphic for responses to IFN-α, IFN-ω, and IFN-β, whereas the remaining 10 in-frame variants (V96F, P130H, M155I, Y215C, A264, P334L, S340G, S422R, N44del, and P335del) were LOF or hypomorphic for responses to IFN-α and IFN-ω only, with normal or subnormal activity upon stimulation with IFN-β (Fig. 1). These data suggest that missense variants and single-amino acid deletions can affect responses to all type I IFNs tested (W73C, C79R, C79Y, I144K, and S316P), or selectively impair responses to IFN-α and IFN-ω, with responses to IFN-β being completely or partially preserved (V96F, P130H, M155I, Y215C, A264, P334L, S340G, S422R, N44del, and P335del).

### Location of the experimentally deleterious IFNAR1 mutants relative to the ligand binding interface

**Figure 2.**
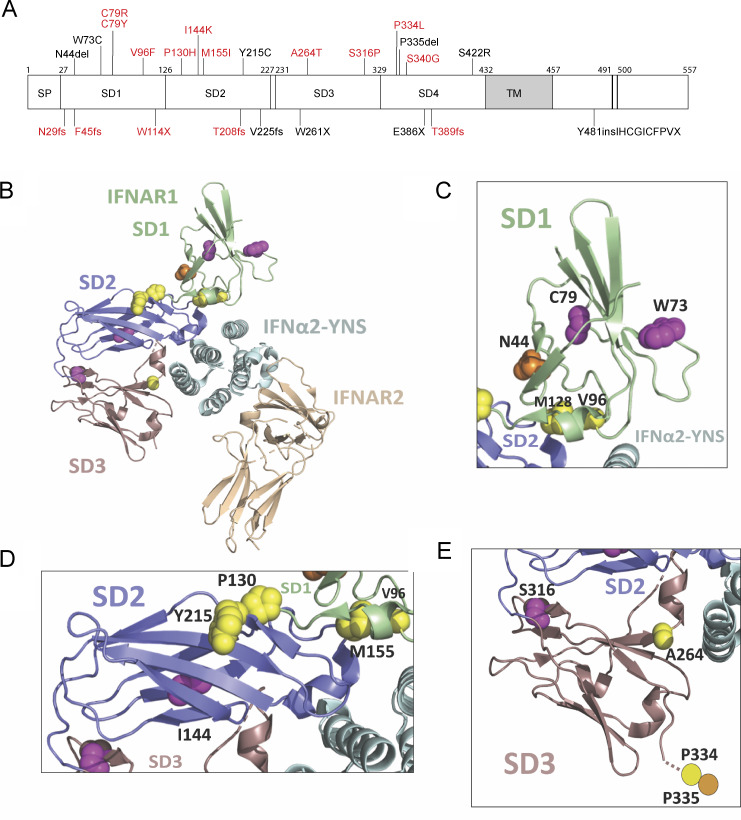
**Location of variants on human IFNAR1. (A)** Schematic representation of full-length IFNAR1 protein, including the four fibronectin type III subdomains (SD1–4), the signal peptide (SP), and the transmembrane domain (TM). The mutations investigated in this study are depicted on the diagram. The hitherto unknown mutations are indicated in red, and the previously reported mutations are indicated in black. **(B)** Ribbon representation of the overall structure of the IFNα2–YNS–IFNAR1–IFNAR2 ternary complex (PDB 3SE3 [Thomas et al., 2011], visualized with PyMOL [version 2.5.5]) showing IFNAR1 with SD1 colored in green, SD2 in blue, and SD3 in violet. IFNα2–YNS is depicted in cyan and IFNAR2 in beige. The amino-acid variants of IFNAR1 described here are highlighted by the depiction of their side chains as spheres across IFNAR1 SD1-3. Variants resulting from a missense mutation are depicted with side chains in yellow. Variants resulting in a complete LOF for IFN-α2, IFN-ω, and IFN-β signaling or hypomorphic for such signaling are depicted with side chains in magenta. In-frame indel variants are depicted with side chains in orange. Variants resulting from a frameshift mutation or an early stop codon (F45fs, W114X, T208fs, V225fs, and W261X) are not shown. **(C)** Close-up view of IFNAR1 SD1 (green) showing the location of variants N44 (orange), W73, and C79 (both magenta), along with V96 and the adjacent M128 from IFNAR1 SD2 (yellow). The locations of SD1 in relation to IFNAR1 SD2 (blue) and IFNα2-YNS (cyan) are shown. **(D)** Close-up view of IFNAR1 SD2 (blue), showing the location of P103, M128, and Y215 variants and the adjacent V96 from IFNAR1 SD1 (yellow), together with I144 (magenta). The locations of IFNAR1 SD2 (slate blue) in relation to IFNAR1 SD1 (green), IFNAR1 SD3 (violet), and IFNα2-YNS (cyan) are shown. **(E)** Close-up view of IFNAR1 SD3 (violet) showing the locations of the variants A264 (yellow) and S316 (magenta). The approximate locations of P334 (yellow circle) and P335 (orange circle), which were not resolved in the IFNα2–YNS–IFNAR1–IFNAR2 crystal structure, are predicted. The locations of IFNAR1 SD3 (violet) in relation to IFNAR1 SD2 (blue) and IFNα2-YNS (cyan) are shown.

We visualized the location of the variants on IFNAR1, displaying the amino-acid side chains of the LOF, hypomorphic, missense, and in-frame indel variants as spheres on the IFNAR1 chain in the IFN-α2-YNS ternary complex (Fig. 2, A and B) (Thomas et al., 2011). Close-up views of IFNAR1 subdomains (SD) 1–3 revealed the location of the residues of the side chains of the variants relative to IFNα2-YNS and the other SD resolved in the published structure (IFNAR1 SD4 was not resolved in the structure and is therefore not displayed) (Fig. 2, C–E). On IFNAR1 SD1, N44, W73, and C79 are located distal to the ligand-binding site whereas V96 is adjacent to M155 on IFNAR1 SD2 and the IFN (Fig. 2 C). On IFNAR1 SD2, P130, I144, and Y215 are distal to the ligand-binding site whereas M155 is adjacent to V96 on IFNAR1 SD1 and the IFN (Fig. 2 D). On IFNAR1 SD3, A264 is located adjacent to the IFN whereas S316 is distal to the ligand-binding site (Fig. 2 E). These analyses suggest that some of the substituted residues on IFNAR1 might directly influence binding to IFNs, whereas others more distal to the ligand-binding interface may affect IFNAR1 function indirectly, possibly by altering receptor conformation. These results led us to investigate further the expression and response of these variants to all IFN subtypes and to glycosylated and non-glycosylated IFNs.

### Expression of IFNAR1 mutant proteins in vitro

**Figure 3.**
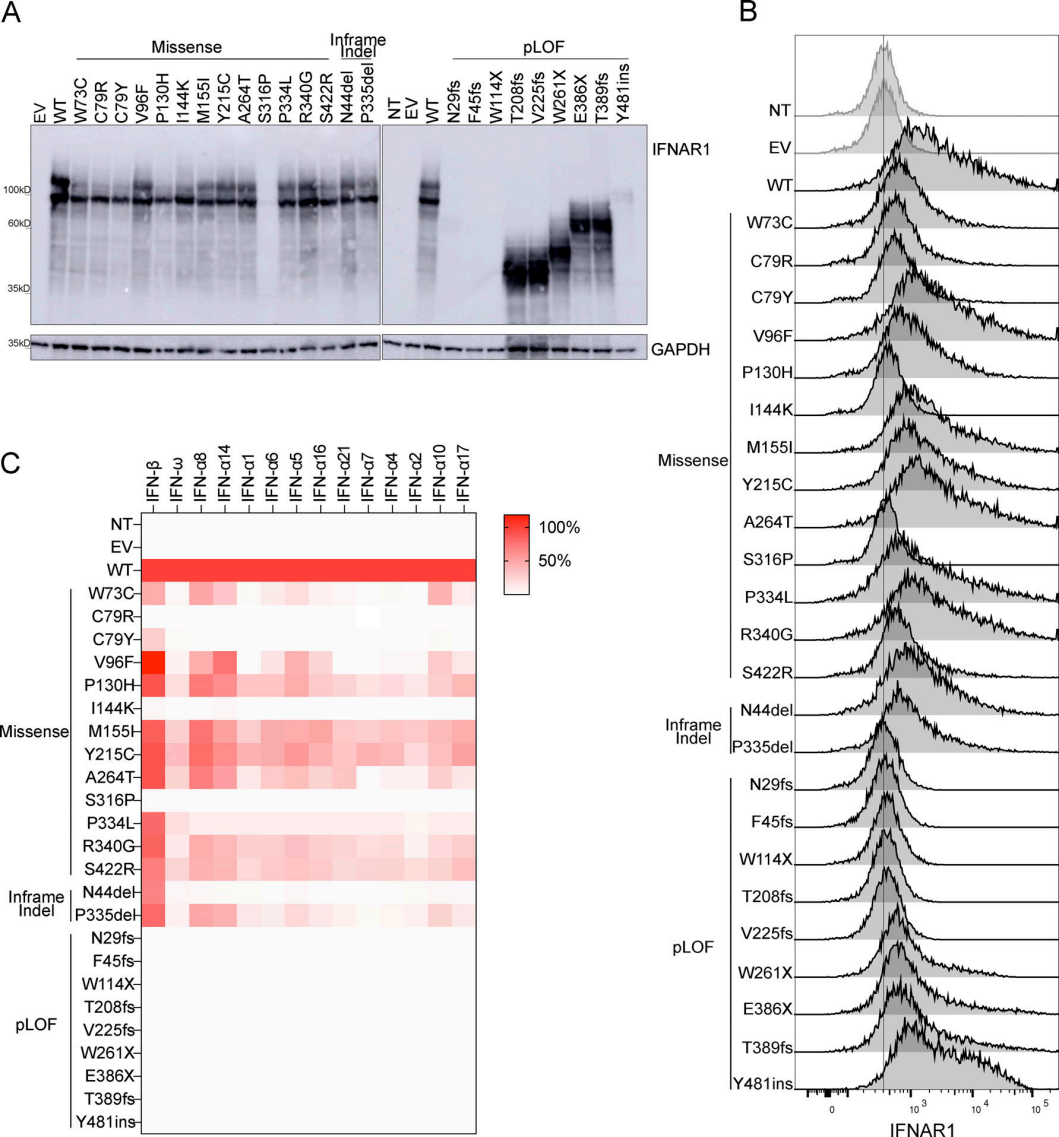
**Expression of *IFNAR1* variants and their impact on the response to type I IFNs. (A)** Western blotting for IFNAR1 in IFNAR1-deficient HEK293T cells transiently transfected with WT or mutant IFNAR1 cDNA constructs. An antibody recognizing the N-terminus of the IFNAR1 protein was used. GAPDH was used as a loading control. A representative blot from at least two experiments is shown. NT, non-transfected; EV, empty vector. **(B)** Flow cytometry histogram of cell-surface IFNAR1 levels in IFNAR1-deficient HEK293T cells transiently transfected with WT or mutant IFNAR1 cDNA constructs and then subjected to extracellular staining with a specific antibody recognizing the N-terminal part (SD2) of the IFNAR1 protein. All histogram plots are representative of at least two independent experiments. **(C)** IFNAR1-deficient HEK293T cells were transiently transfected with WT or mutant IFNAR1 cDNA constructs and were then stimulated with the indicated IFNs for 24 h, and luciferase activity was measured. The IFN-α subtypes are arranged in order of affinity for IFNAR1 binding, from the highest (left, IFN-α8) to the lowest (right, IFN-α17) affinity (Table S1). The heatmap shows the mean luciferase activity relative to the WT from two independent experiments. Source data are available for this figure: SourceData F3.

We investigated IFNAR1 expression following the transient transfection of IFNAR1-deficient HEK293T cells—generated by CRISPR/Cas9-mediated gene editing—with plasmids encoding the variant IFNAR1 proteins. Western blotting with an antibody specific for the N-terminal region of IFNAR1 showed that the proteins encoded by most of the missense (W73C, C79R, C79Y, V96F, P130H, I144K, M155I, Y215C, A264, P334L, S340G, and S422R), and single-amino acid deletion (P335del and N44del) variants were produced normally in our overexpression system. Western blotting showed that the S316P variant was loss-of-expression (Fig. 3 A). The pLOF variants yielded truncated proteins, migrating at a molecular weight below that of the WT IFNAR1 (T208fs, V225fs, W261X, E386X, T389fs, and Y481insIHCGICFPV*) or resulted in a loss of expression (N29fs, F45fs, and W114X) (Fig. 3 A). Two smeary bands were obtained for both the WT and IFNAR1 mutant proteins on a western blot of cell extracts (Fig. 3 A). Treatment with PNGase F led to the detection of bands with a lower molecular weight (MW), indicating that the higher MW bands represented glycosylated forms of IFNAR1 (Fig. S1 B). This result suggests that mutant IFNAR1 proteins may affect the IFNAR1 glycosylation process. We then used flow cytometry to assess the cell-surface expression of the variants in the same overexpression system. Some missense proteins (V96F, P130H, M155I, Y215C, A264T, P334L, P340G, and N44del) were normally expressed on the cell plasma membrane, whereas others were poorly expressed (W73C, C79R, C79Y, P335del, and S422R) or were not detected at all on the plasma membrane (I144K and S316P) (Fig. 3 B). The pLOF proteins were not detected on the plasma membrane (N29fs, F45fs, W114X, T208fs, and V225fs), or were detected in only trace amounts, probably due to the overexpression of intronless plasmids (W261X, E386X, and T389fs) (Fig. 3 B). Y481insIHCGICFPV* was expressed at the cell surface, as previously reported (Bastard et al., 2021b). In summary, overexpression of the mutant IFNAR1 proteins revealed different patterns of total protein production and expression at the cell surface, suggesting different impacts on the responses of these variants to type I IFNs.

### Response of IFNAR1 mutant proteins to type I IFNs

**Figure S2.**
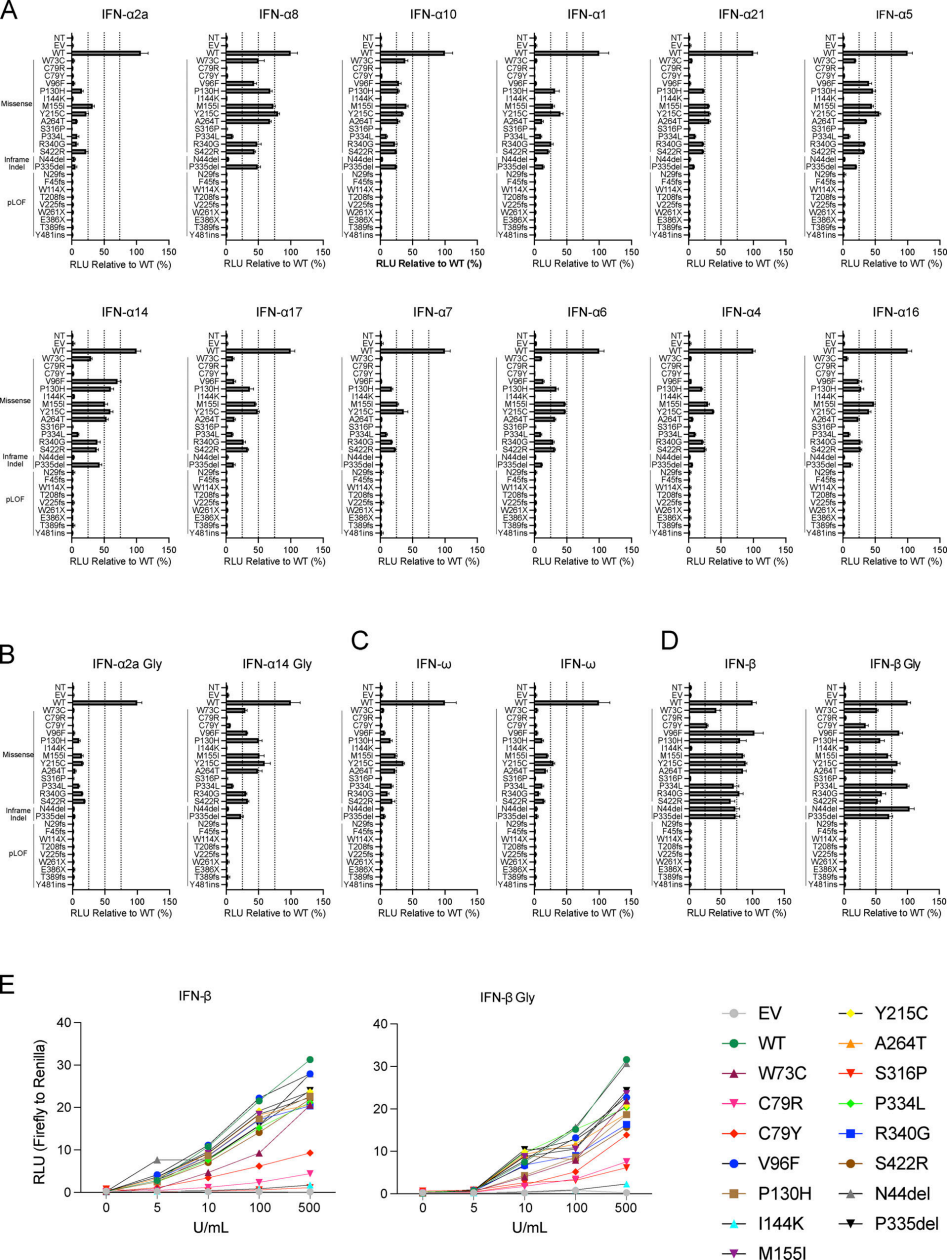
**Functional characterization of IFNAR1 variants in terms of the response to type I IFNs. (A–D)** IFNAR1-deficient HEK293T cells transiently transfected with WT or mutant IFNAR1 cDNA constructs were stimulated with IFN-α subtypes (1,000 U/ml, A), glycosylated IFN-α2a or IFN-α14 (1,000 U/ml, B), non-glycosylated or glycosylated IFN-ω (1 ng/ml, C), or non-glycosylated or glycosylated IFN-β (100 U/ml) for 24 h, and luciferase activity was measured relative to WT. **(E)** Luciferase signal readings across a range of titrated concentrations of non-glycosylated or glycosylated IFN-β. The graphs show the mean ± SEM of two independent experiments.

We then investigated the impact of these *IFNAR1* variants on cellular responses to the different type I IFNs. We stimulated the cells with the 12 IFN-α subtypes, IFN-ω, or IFN-β in a luciferase assay. We did not stimulate the cells with IFN-κ or -ɛ because these cytokines have a low affinity for the IFN receptor, and their expression is restricted to the skin and uterus, respectively. As expected, none of the pLOF variants responded to any of the IFNs tested (Fig. 3 C and Fig. S2, A–D). Missense and in-frame-indel variants displayed various patterns of response to IFN-α subtypes and IFN-ω. C79R, C79Y, I144K, S316P, and N44del were LOF in response to all IFN-α subtypes and IFN-ω, whereas the other variants were hypomorphic with some, but not all IFN-α subtypes and IFN-ω. The responses to IFN-α8 and IFN-α14—the IFN-α subtypes with the highest affinity for IFNAR1 (Table S1) (Wittling et al., 2021)—gave the strongest luciferase signals for variants in the hypomorphic range. Upon stimulation with IFN-β, C79R, I144K, and S316P were LOF, whereas W73C and C79Y were hypomorphic (Fig. 3 C; and Fig. S2, D and E). However, all the other variants, which were LOF or hypomorphic for responses to IFN-α subtypes and IFN-ω, had normal responses to IFN-β (>50% of WT signal) (Fig. 3 C and Fig. S2, A–E). In humans, IFN-α2, IFN-α14, IFN-β, and IFN-ω are normally glycosylated. We therefore investigated the response of the variants to glycosylated IFNs. We found no overall difference in the response to glycosylated and non-glycosylated forms of IFN-α2, IFN-α14, IFN-β, and IFN-ω (Fig. 3 C and Fig. S2, B–E). Together, our data showed that the V96F, P130H, M155I, Y215C, A264, P334L, S340G, S422R, N44del, and P335del in-frame IFNAR1 mutant proteins resulted in dissociated cellular responses to type I IFNs, with impaired cellular responses to all IFN-α subtypes (except IFN-α8 and IFN-α14) and IFN-ω but not IFN-β, whereas the W73C, C79R, C79Y, I144K, and S316P variants affected responses to all IFNs equally.

### Dominant-negative *IFNAR1* variants

**Figure 4.**
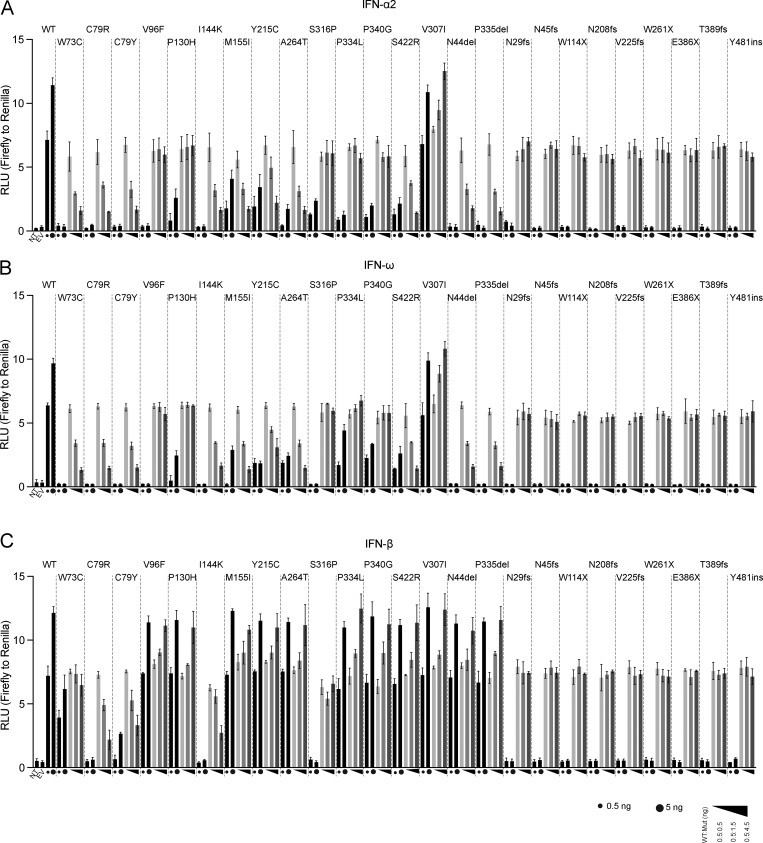
**Negative dominance assay for *IFNAR1* variants. (A–C)** IFNAR1-deficient HEK293T cells cotransfected with luciferase reporter plasmids plus EV (up to 15 ng) and various amounts of plasmids encoding WT and/or variant IFNAR1 (0.5, 1.5, 4.5, and 5 ng). The amount of plasmid used for transfection (ng) is indicated in the figure. Cells were stimulated with IFN-α2 (A, 1,000 U/ml), IFN-ω (B, 1 ng/ml), or IFN-β (C, 100 U/ml) for 24 h, and luciferase activity was measured. Graphs depict the mean ± SEM of two independent experiments.

We then investigated whether the mutant IFNAR1 proteins exerted a dominant-negative effect on the WT IFNAR1 protein. We performed an ISRE luciferase assay in which *IFNAR1*-deficient HEK293T cells were transfected with various amounts of WT or IFNAR1 mutant plasmids alone, or cotransfected with WT IFNAR1 together with various amounts of the mutant variants. In addition to the deleterious *IFNAR1* variants, we randomly selected the neutral V307I variant as a control in our experiments. As expected, the strength of the luciferase signal increased with increasing amounts of WT or V307I IFNAR1, or a combination of WT and V307I IFNAR1, following stimulation with IFN-α, IFN-ω, and IFN-β (Fig. 4). Upon stimulation with IFN-α or IFN-ω, increasing amounts of W73C, C79R, C79Y, I144K, M155I, Y215C, A264T, S422R, N44del, and P335del variants were consistently associated with lower levels of luciferase activity. Other missense variants, including V96F, P130H, S316P, P334L, and P349G, and all the pLOF variants displayed no interference with the WT IFNAR1 signaling following stimulation with IFN-α or IFN-ω (Fig. 4, A and B). Following stimulation with IFN-β, increasing amounts of C79R, C79Y, and I144K only were associated with lower levels of luciferase activity, whereas no interference with WT IFNAR1 signaling was observed for the S316P variant or any of the pLOF variants (Fig. 4 C). Consistent with our earlier findings, none of the other *IFNAR1* variants had any impact on WT IFNAR1 activity, resulting in a gradual increase in luciferase levels in response to stimulation with IFN-β (Fig. 4 C). Collectively, our results indicate that seven variants are dominant-negative for cellular responses to IFN-α and IFN-ω only (W73C, M155I, Y215C, A264T, S422R, N44del, and P335del), whereas three variants are dominant-negative for responses to IFN-α, IFN-ω, and IFN-β (C79R, C79Y, I144K) (Table S2). This is intriguing as the negative dominance of these variants is not accompanied by an increase in the expression of the variant at the surface of the cell due to a loss of the recycling motif, as seen, for example, with IFNGR1 (Jouanguy et al., 1999) and IL6ST (Béziat et al., 2020). These findings suggest that the mutant proteins disrupt the function of WT proteins by interfering with their activity and that this interference also depends on their heterodimerization with IFNAR2. This implies that the IFNAR1 and IFNAR2 receptor complexes may self-assemble or undergo higher-order oligomerization.

### Expression and function of IFNAR1 variants in the patients’ fibroblasts

**Figure 5.**
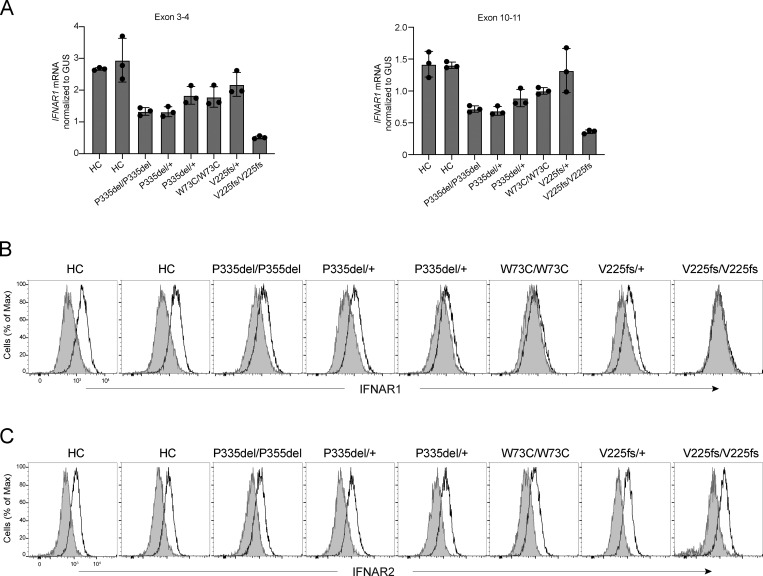
**Expression of IFNAR1 by the patients’ fibroblasts. (A)** IFNAR1 mRNA levels in SV40-fibroblasts from two healthy controls (C1, C2), and patients with IFNAR1 variants: P335del/P335del, P335del/+, W73C/W73C, V225fs/+, and V225fs/V225fs. GUS was used as an expression control. Graphs depict the mean ± SEM of two independent experiments, each with three technical duplicates. **(B and C)** Flow cytometry histograms of cell-surface expression for IFNAR1 (B) and IFNAR2 (C), with extracellular staining of SV40-fibroblasts from healthy controls and patients. Antibodies recognize the extracellular parts of IFNAR1 or IFNAR2. Each histogram plot is representative of two independent experiments.

**Figure 6.**
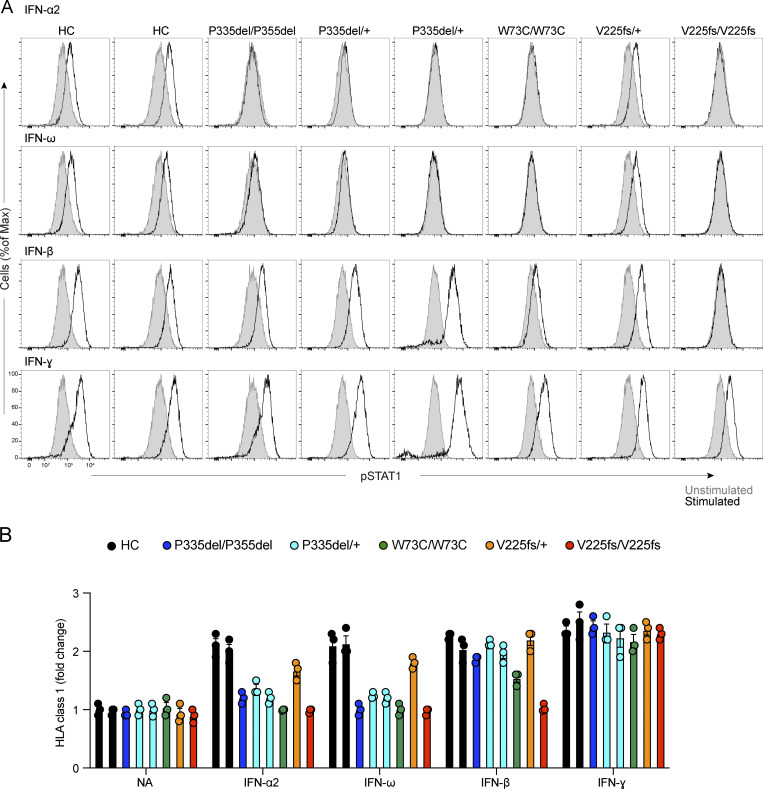
**Function of IFNAR1 variants in the patients’ fibroblasts. (A)** Intracellular FACS staining of phosphorylated STAT1 in SV40-fibroblasts stimulated with IFN-α2a (1,000 U/ml), IFN-ω (1 ng/ml), IFN-β (100 U/ml), or IFN-γ (1,000 U/ml) for 15 min, for two healthy controls and patients with IFNAR1 variants. **(B)** Fold-change in HLA class I levels analyzed by flow cytometry with extracellular staining in SV40-fibroblasts stimulated with IFN-α2a, IFN-ω, IFN-β, or IFN-γ for 48 h. Graphs depict the mean ± SEM of two independent experiments.

**Figure S3.**
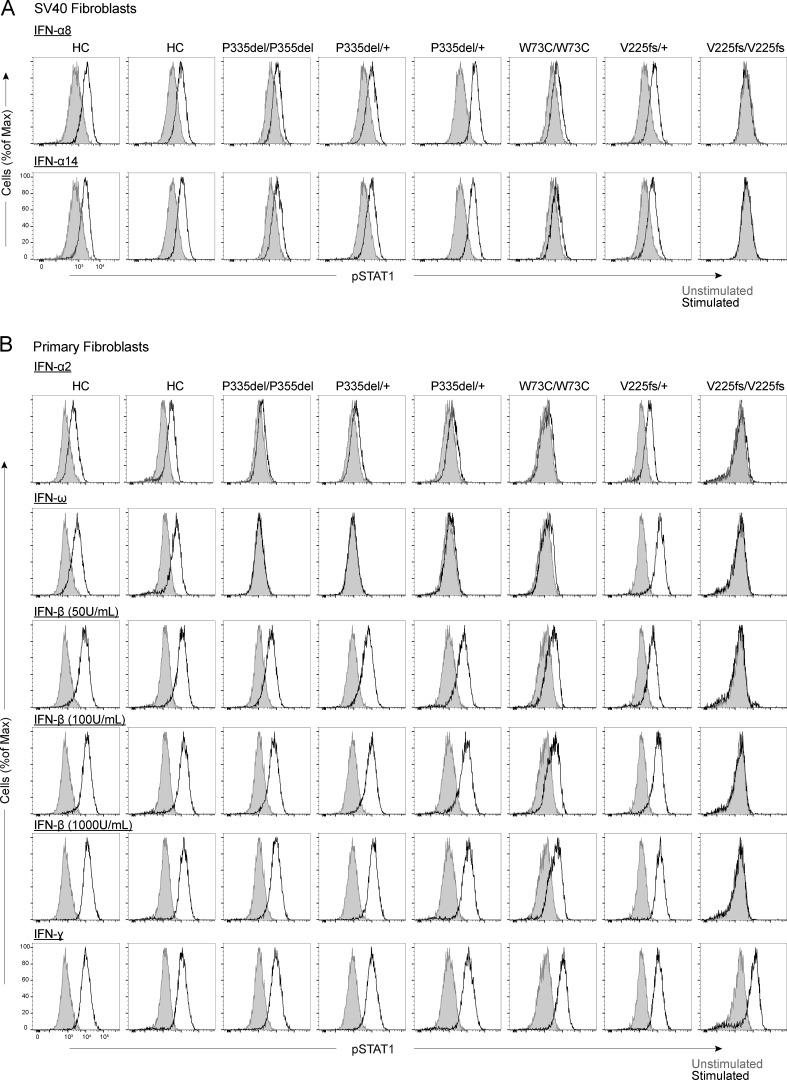
**Function of IFNAR1 variants in the patients’ fibroblasts. (A)** Intracellular FACS staining of phosphorylated STAT1 in SV40 fibroblasts stimulated with IFN-α8 (1,000 U/ml) or IFN-α8 (1,000 U/ml) for 15 min for two healthy controls and patients. **(B)** Intracellular FACS staining of phosphorylated STAT1 in primary fibroblasts stimulated with IFN-α2a (1,000 U/ml), IFN-ω (1 ng/ml), IFN-β (50, 100, and 100 U/ml), or IFN-γ (1,000 U/ml) for 15 min, for two healthy controls and patients. The graphs show representative data from two independent experiments.

We then generated and tested SV40-transformed dermal fibroblasts from individuals homozygous or heterozygous for P335del, homozygous for W73C, heterozygous or homozygous for V225fs, and healthy controls. Reverse transcription and quantitative real-time PCR (RT-qPCR) showed that IFNAR1 mRNA levels were normal to moderately decreased in P335del/P335del, P335del/+, W73C/W73C, and V225fs/+ cells, contrasting with very low levels in V225fs/V225fs cells, relative to healthy controls (Fig. 5 A). We used flow cytometry to assess the surface expression of IFNAR1 in the patients’ cells. IFNAR1 levels on the cell surface were lower in P335del/P335del and P335del/+ cells, much lower in W73C/W73C cells, and normal in V225fs/+ cells, and no IFNAR1 was detected on the surface of V225fs/V225fs cells (Fig. 5 B). IFNAR2 levels were normal in all cells (Fig. 5 C). We studied the responses of SV40 fibroblasts to stimulation with IFN-α2, -β, and -ω. Consistent with the data for HEK293T cells, the stimulation of SV40-fibroblasts with IFN-α2a or IFN-ω for 15 min did not induce the phosphorylation of STAT1 in P335del/P335del, P335del/+, or W73C/W73C cells (Fig. 6 A). By contrast, P335del/P335del, P335del/+, and W73C/ W73C cells displayed STAT1 phosphorylation in response to stimulation with IFN-β at levels similar to those in healthy control cells (Fig. 6 A). We then assessed the responses of SV40 fibroblasts to IFN-α8 and IFN-α14, the IFN-α subtypes with the highest affinity for IFNAR1. Consistently, STAT1 phosphorylation was induced normally in P335del/P335del and P335del/+ cells but was markedly decreased in W73C/W73C cells after stimulation with IFN-α8 and IFN-α14 (Fig. S3 A). V225fs/+ cells displayed STAT1 phosphorylation in response to stimulation with IFN-α, -β, and -ω and, as previously described, STAT1 phosphorylation in response to IFN-α, -β, and -ω was abolished in IFNAR1-deficient V225fs/V225fs fibroblasts (Fig. 6 A and Fig. S3 A). By contrast, STAT1 phosphorylation in response to IFN-γ stimulation was normal in all cells (Fig. 6 A). Similar results were obtained in primary fibroblasts and with different concentrations of IFN-β (Fig. S3 B). We then assessed the late responses of the patients’ cells to IFN-α2, -β, and -ω by measuring HLA class I induction 48 h after stimulation with IFNs. Control cells displayed an increase in HLA class I expression (approximately twofold) after stimulation with IFN-α and IFN-ω (Fig. 6 B). Consistent with the STAT1 phosphorylation results, P335del/P335del, P335del/+, and W73C/W73C cells displayed no induction of HLA class I expression after stimulation with IFN-α2 and -ω, whereas HLA class I induction in response to stimulation with IFN-β was normal (Fig. 6 B). V225fs/+ cells displayed an increase in HLA class I expression in response to IFN-α2, -β, and -ω, whereas IFNAR1-deficient V225fs/V225fs cells displayed no HLA class I induction after stimulation with IFN-α2, -β, and -ω, and the response to IFN-γ stimulation was normal in all cells (Fig. 6 B). These findings reveal the different impacts of *IFNAR1* genotypes on cellular responses to various type I IFNs, highlighting the specific impairment of low-affinity IFN-α (excluding IFN-α8 and IFN-α14) and IFN-ω signaling in *IFNAR1* P335del/P335del, P335del/+, and W73C/W73C cells, in which IFN-β signaling remains largely intact.

### Susceptibility of the patients’ fibroblasts to SARS-CoV-2 in the presence or absence of IFNs

**Figure 7.**
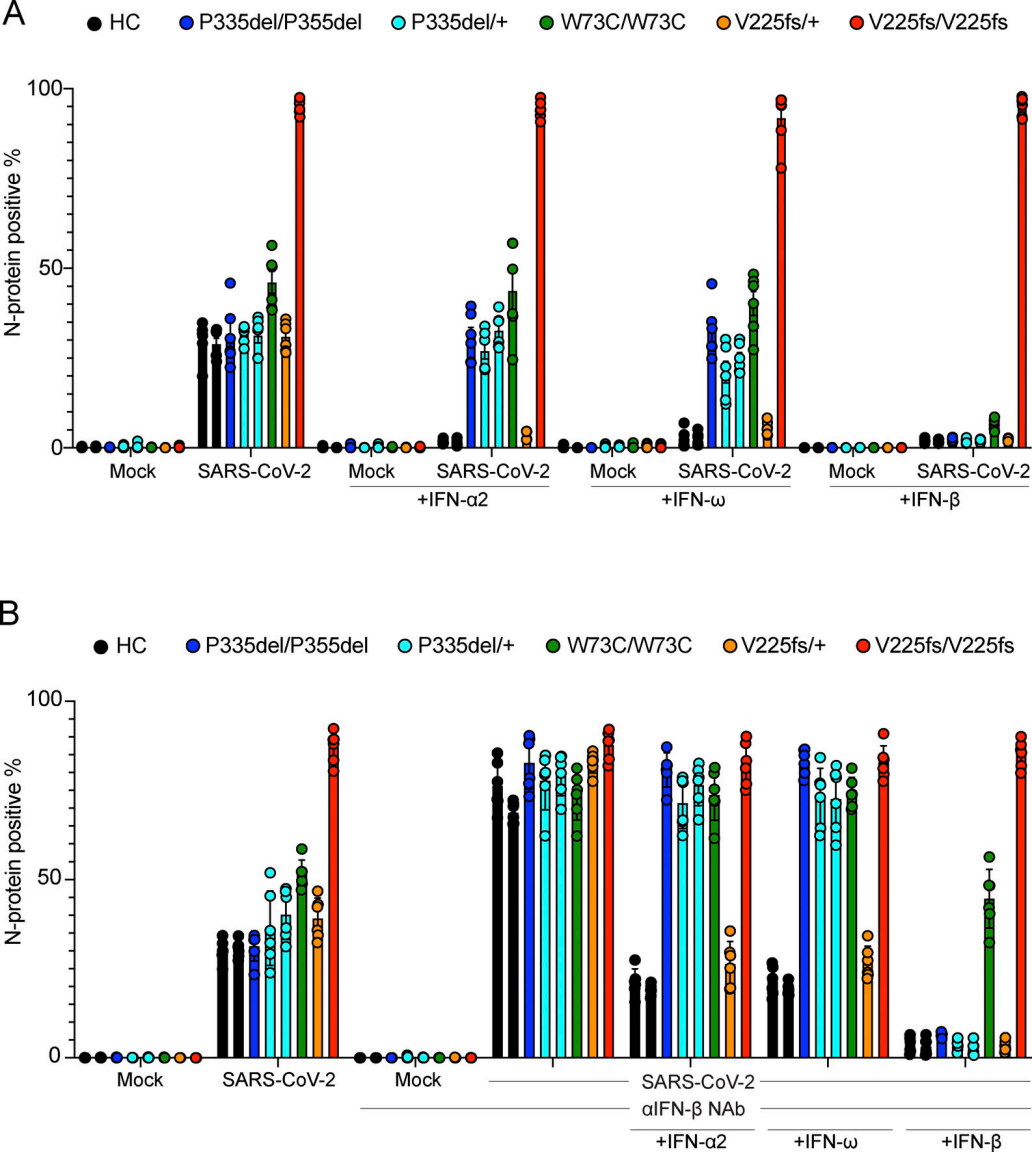
**SARS-CoV-2 infection in the cells of an IFNAR1-deficient patient in vitro. (A)** Immunofluorescence (IF) analysis for the SARS-CoV-2 N protein in SV40-fibroblasts from healthy controls (C1 and C2) and patients with IFNAR1 variants including P335del/P335del, P335del/+ (two patients), W73C/W73C, V225fs/+, and V225fs/V225fs. Cells were treated with IFN-α2a (100 U/ml), IFN-ω (1 ng/ml), or IFN-β (10 U/ml) overnight before infection with SARS-CoV-2 (MOI = 0.5). Cells were fixed and stained 48 h after infection. **(B)** IF analysis for the SARS-CoV-2 N protein in SV40-fibroblasts treated with neutralizing anti-IFN-β antibodies. Cells were treated with anti-IFN-β neutralizing antibodies and then with IFN-α2a (100 U/ml), IFN-ω (1 ng/ml), or IFN-β (100 U/ml) overnight. They were then infected with SARS-CoV-2 infection (MOI = 0.5). Cells were fixed and stained 48 h after infection. Graphs depict the mean ± SEM of two or three independent experiments.

**Figure S4.**
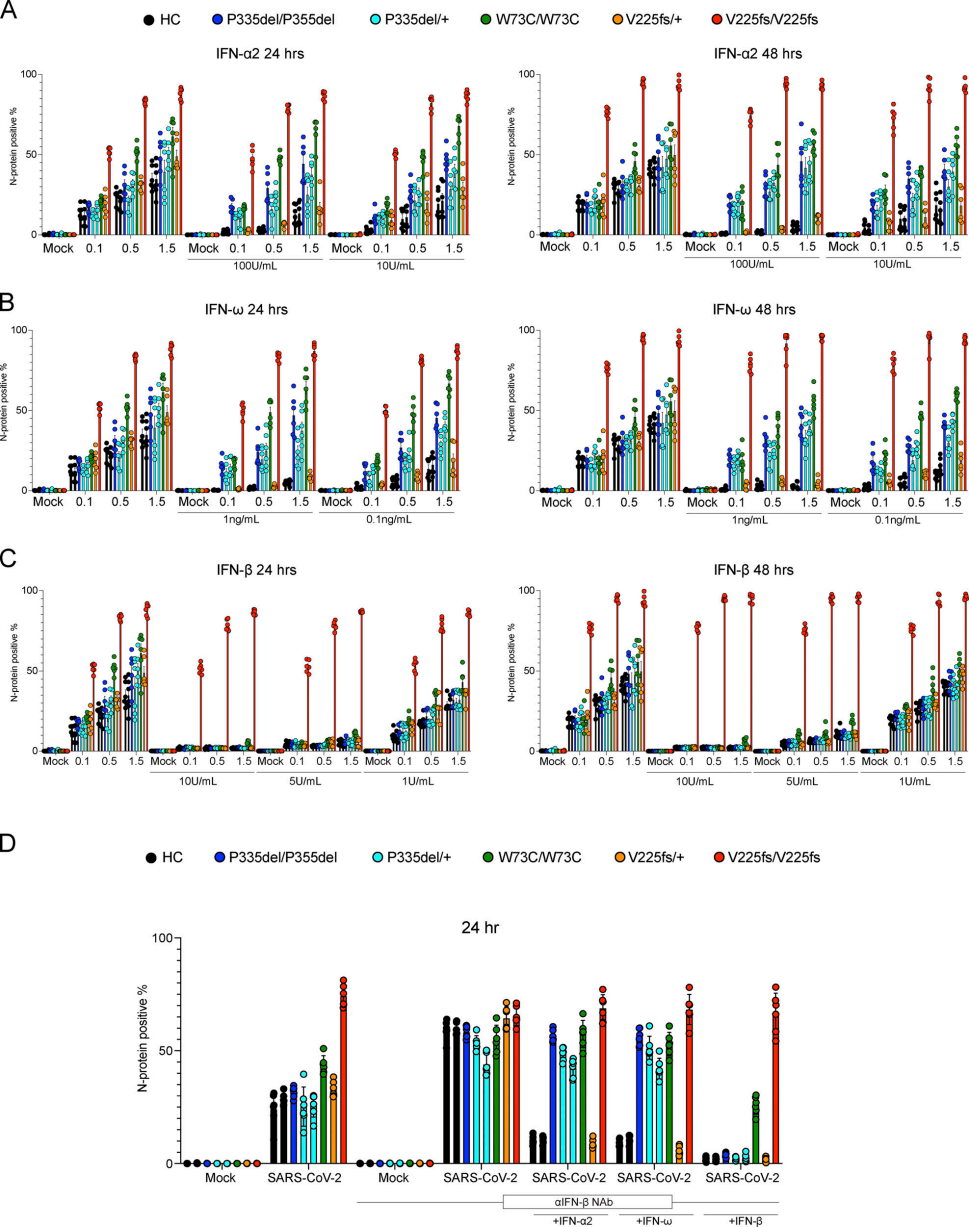
**SARS-CoV-2 infection of IFNAR1-deficient patient cells in vitro. (A–C)** IF analysis for the SARS-CoV-2 N protein in SV40-fibroblasts from healthy controls (C1 and C2) and patients with IFNAR1 variants including P335del/P335del, P335del/+ (two patients), W73C/W73C, V225fs/+, and V225fs/V225fs. Cells were treated with IFN-α2a (100 or 10 U/ml, A), IFN-ω (1 or 0.1 ng/ml, B), or IFN-β (10, 5, or 1 U/ml, C) overnight and then infected with SARS-CoV-2 at MOI = 0.1, 0.5, or 1.5. Cells were fixed and stained 24 or 48 h after infection. **(D)** IF analysis for the SARS-CoV-2 N protein in SV40-fibroblasts treated with neutralizing antibodies against IFN-β then stimulated with IFN-α2a (100 U/ml), IFN-ω (1 ng/ml), or IFN-β (100 U/ml). Cells were then infected with SARS-CoV-2 at MOI = 0.5. Cells were fixed and stained 24 h after infection. The graphs depict the mean ± SEM of two or three independent experiments.

We assessed the cellular impact of the *IFNAR1* genotypes (P335del/P335del, P335del/+, W73C/W73C, V225fs/+, and V225fs/V225fs) on cellular antiviral activity. We evaluated SARS-CoV-2 replication in patient-specific SV40-fibroblasts that had been transduced with angiotensin-converting enzyme 2 (ACE2) to facilitate viral entry, rendering them susceptible to SARS-CoV-2 infection. IFNAR1-deficient V225fs/V225fs cells displayed high levels of SARS-CoV-2 infection. For all the other IFNAR1 variants, the proportions of SARS-CoV-2-infected cells were similar to those for control cells at 24 and 48 h and various multiplicities of infection (MOI) (Fig. 7 A and Fig. S4, A–C). SARS-CoV-2 infection levels appeared to be higher in W73C/W73C cells, but remained lower than that in IFNAR1-deficient cells, consistent with our finding that W73C was hypomorphic in response to IFN-β. We then investigated whether treatment with IFN-α, -ω, or -β could inhibit SARS-CoV-2 replication. Treatment with IFN-α or -ω did not inhibit SARS-CoV-2 replication in P335del/P335del, P335del/+, or W73C/W73C cells at 24 or 48 h at various MOI (Fig. 7 A and Fig. S4, A–C). V225fs/+ cells responded to treatment with IFN-α or -ω and were able to control SARS-CoV-2 replication, ruling out IFNAR1 haploinsufficiency (Fig. 7 A and Fig. S4, A–C). By contrast, IFN-β treatment inhibited SARS-CoV-2 replication in all cells, including P335del/P335del, P335del/+, and W73C/W73C cells at 24 or 48 h and with various IFN concentrations and viral MOI (Fig. 7 A and Fig. S4, A–C). As expected, IFNAR1-deficient V225fs/V225fs cells did not respond to treatment with IFN-α, -ω, or -β and did not limit SARS-CoV-2 infection (Fig. 7 A and Fig. S4, A–C). Human fibroblasts constitutively produce basal levels of bioactive IFN-β (Gao et al., 2021). We therefore used neutralizing antibodies against IFN-β to eliminate the effect of this basal IFN-β in similar SARS-CoV-2 infection experiments. Following IFN-β neutralization, all cells had high levels of SARS-CoV-2 infection, similar to those in IFNAR1-deficient V225fs/V225fs cells (Fig. 7 B and Fig. S4 D). We then investigated whether treatment with IFN-α or -ω in the presence of neutralizing anti-IFN-β antibodies inhibited SARS-CoV-2 replication. Consistent with our previous results, treatment with IFN-α or -ω did not inhibit SARS-CoV-2 replication in P335del/P335del, P335del/+, or W73C/W73C cells at 24 or 48 h, as shown by comparison with control or V225fs/+ cells, whereas it did inhibit the replication of the virus in cells with other genotypes (Fig. 7 B and Fig. S4 D). We then added excess IFN-β following IFN-β neutralization. P335del/P335del and P335del/+ cells completely restricted SARS-CoV-2 infection, and W73C/W73C cells were partially protected against SARS-CoV-2 infection, again consistent with W73C being hypomorphic in terms of the response to IFN-β (Fig. 7 B and Fig. S4 D). Thus, heterozygosity (P335del) or homozygosity (P335del and W73C) for *IFNAR1* variants impairs type I IFN immunity to SARS-CoV-2 infection in cells stimulated with IFN-α and IFN-ω.

### Life-threatening viral diseases in several unrelated patients

**Figure S5.**
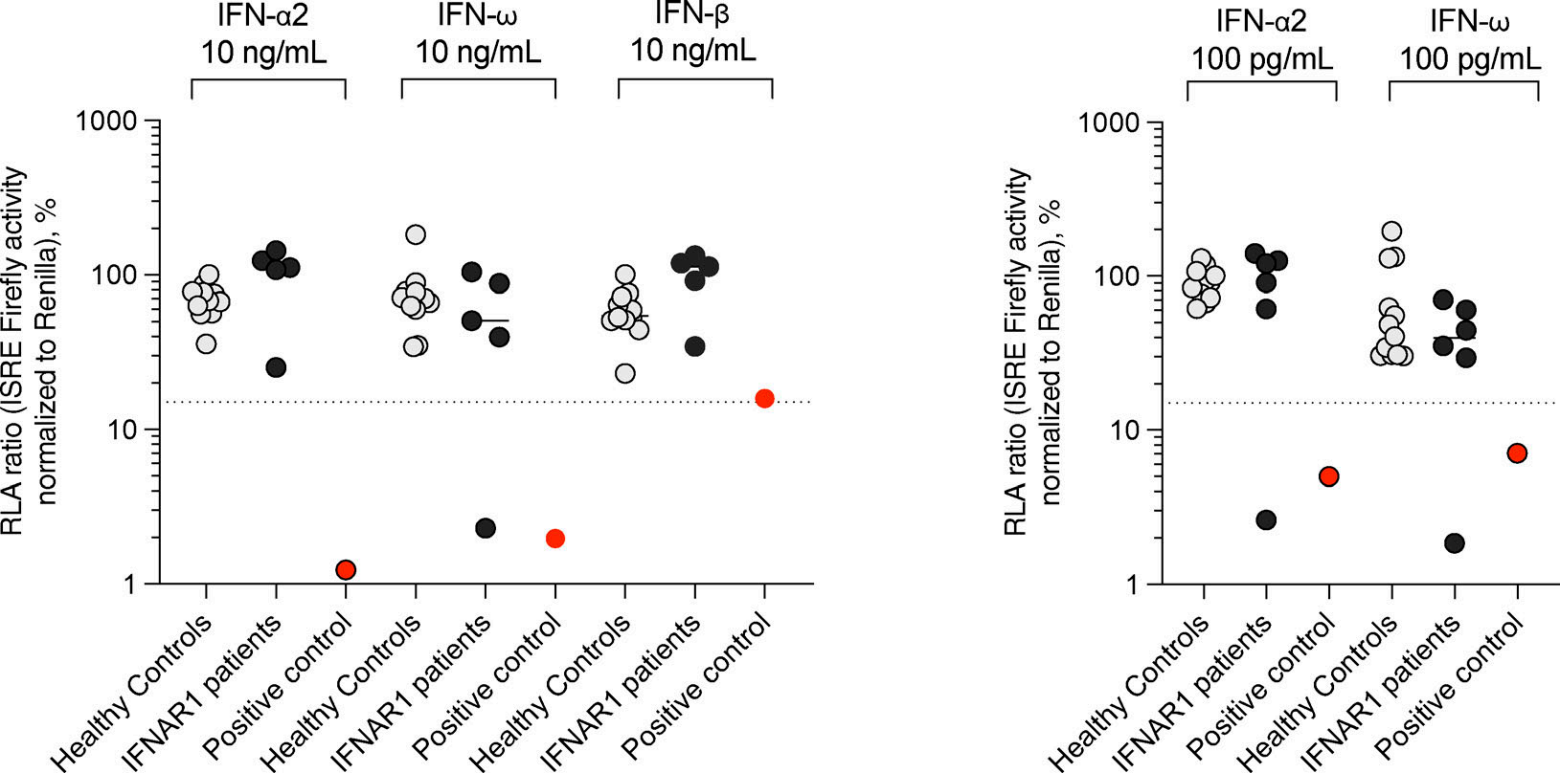
**Auto-Abs neutralizing type I IFNs in the patients with IFNAR1 variants.** Luciferase-based neutralization assays for detecting auto-Abs neutralizing 10 ng/ml IFN-α2, IFN-ω, or IFN-β (left panel) and 100 pg/ml IFN-α2 or IFN-ω (right panel). Plasma samples from healthy controls (gray), patients with IFNAR1 variants (black; P1, 3, 6, 9, 17, and 27), and an APS-1 patient (red, positive control) were diluted 1:10. HEK293T cells were cotransfected with a plasmid containing the firefly luciferase gene under the control of an IFN-sensitive response element (ISRE)-containing promotor and a plasmid containing the *Renilla* luciferase gene. The cells were then treated with type I IFNs, and relative luciferase activity (RLA) was calculated by normalizing firefly luciferase activity against *Renilla* luciferase activity. An RLA <15% of the value for the mock treatment was considered to correspond to neutralizing activity (dotted line; Bastard et al., 2021a).

The clinical profiles of 11 individuals heterozygous for dominant-negative *IFNAR1* variants revealed susceptibility to a broad spectrum of viral illnesses with various degrees of severity (P2–P4, P6, P8, and P10–16). There are also rare homozygotes for these variants (P1, P5, P7, P9, and P17–29). The clinical profiles are described in detail in the supplementary materials. Briefly, these patients have diverse ethnic backgrounds including Arab, Brazilian, Cambodian, Chinese, Indian, Iranian, Israeli, Ivorian, Lebanese, Mexican, Pakistan, Spanish, Turkish, and Western Polynesian, and their ages range from 1 to 84 years (Table 1). They have suffered from severe infections of the respiratory tract (COVID-19) and central nervous system (HSE, Japanese encephalitis virus [JEV] encephalitis, enterovirus 71 [EV71] encephalitis, and adverse reactions to LAV, mostly the MMR and YF vaccines). The biallelic and monoallelic in-frame variants of IFNAR1 are mostly associated with COVID-19 (W73C, C79R, C79Y, M155I, A264T, S422R, and P335del) and encephalitis (Y215C and N44del), whereas AR loss-of-expression IFNAR1 genotypes (N29fs, V225fs, W261X, E386X, T389fs, and Y481insIHCGICFPV*) also increase susceptibility to LAV vaccines in children, particularly for the MMR and YFV vaccines. Clinical outcomes differed considerably between these patients. Some patients survived severe or critical COVID-19 pneumonia (P1, 9, and 11), encephalitis (P5, 8, 15, and 16), or adverse vaccine reactions (P17–19, 24–27, and 29), whereas others succumbed to critical COVID-19 pneumonia (P3, 4, 7, and 12), HSE (P28), or adverse reactions to the MMR vaccine (P20–23). We also analyzed all genes underlying known IEIs present in these patients with IFNAR1 variants (Tangye et al., 2022). P2, who is homozygous for a splicing mutation of IL12RB1, was the only patient homozygous for a pLOF variant (Table S3). Other patients had homozygous missense mutations predicted to be benign, including TYK2 R703W in P16, which was confirmed to be biochemically neutral (Tomasson et al., 2008). We also checked for AD diseases and found no pLOF variants present in the heterozygous state. We identified 24 monoallelic missense mutations, all predicted to be benign (Table S3). We also checked for auto-Abs neutralizing type I IFNs in patients with available plasma or serum samples (P1, 3, 6, 9, 17, and 27). P17 was positive for auto-Abs against IFN-α2a and IFN-ω, but all the other patients were negative for auto-Abs neutralizing type I IFNs (Fig. S5).

**Table 1.** *IFNAR1* deleterious variants in patients with life-threatening viral diseases

Category	Patient	Mutation	Origin	Age at presentation and gender	Disease	Outcome	Reference
Missense	P1	W73C/W73C	Turkey	38 years (M)	Critical COVID-19 pneumonia	Survived	Zhang et al. (2020)
	P2	C79R/+	Mexico	4 years (M)	Mild COVID-19	Survived	
	P3	C79Y/+	Ivory coast	63 years (M)	Critical COVID-19 pneumonia	Deceased	
	P4	M155I/+	Lebanon	84 years (M)	Critical COVID-19 pneumonia	Deceased	
	P5	Y215C/Y215C	Spain	2 years (M)	HSE	Survived	Armangué et al. (2023)
	P6	A264T/+	Turkey	9 years (M)	Asymptomatic/mild COVID-19 and frequent respiratory viral infections, including influenza	Survived	
	P7	S422R/S422R	Pakistan	26 years (M)	Critical COVID-19 pneumonia	Deceased	Zhang et al. (2020)
Inframe Indel	P8	N44del/+	Cambodia	2 years (M)	EV71 encephalitis	Survived	
	P9	P335del/P335del	Turkey	17 years (M)	Severe COVID-19 pneumonia	Survived	
	P10	P335del/+	Eastern Asia	30 years (F)	Asymptomatic COVID-19	Survived	
	P11	P335del/+	China	23 years (F)	Critical COVID-19 pneumonia	Survived	Zhang et al. (2020)
	P12	P335del/+	China	57 years (F)	Critical COVID-19 pneumonia	Deceased	
	P13	P335del/+	Thailand	62 years (F)	Critical COVID-19 pneumonia	Survived	
	P14	P335del/+	Hong Kong	32 years (M)	Mild SARS-CoV-2	Survived	
	P15	P335del/+	Cambodia	5 years (M)	JEV encephalitis	Deceased	
	P16	P335del/+	Cambodia	3 years (M)	JEV encephalitis	Survived	
pLOF	P17	N29fs/N29fs	India	2.5 years (F)	Adverse reactions to MMR/V vaccine	Survived	
	P18	V225fs/V225fs	Iran	1 year (M)	Adverse reactions to MMR vaccine	Survived	Hernandez et al. (2019)
	P19	V225fs/W261X	Brazil	12 years (F)	Adverse reactions to YF vaccine	Survived	
	P20	E386X/E386X	West Polynesia	1 year (F)	Adverse reactions to MMR vaccine	Deceased	Bastard et al. (2022a)
	P21			1 year (M)		Deceased	
	P22			15 mo (F)		Deceased	
	P23			13 mo (M)		Deceased	
	P24			16 mo (M)		Survived	
	P25			14 mo (M)	Adverse reactions to MMR vaccine and critical RSV pneumonia	Survived	
	P26			10 mo (F)	Hemophilus influenza type B (Hib) bacteremia and meningitis at the age of 10 mo, critical RSV pneumonia at the age of 12 mo, and ARDS at the age of 7 years	Survived	
	P27	T389fs/T389fs	Israel	1 year (M)	Adverse reactions to MMR/V vaccine	Survived	
	P28	Y481insIHCGICFPV*/Y481insIHCGICFPV*	Palestine	13 mo (F)	HSE	Deceased	Bastard et al. (2021b)
	P29			6 mo (M)	Meningitis at the age of 6 and 10 mo, parotitis and deafness age of 14 years after Mumps infection?	Survived	

### Clinical genetics of P335del *IFNAR1*

With the exception of the E386X null allele of *IFNAR1*, which is common in Western Polynesians (MAF = 0.0125), non-synonymous *IFNAR1* variants are globally rare (MAF < 0.0001). However, P335del *IFNAR1* is common in Southern China. The overall allele frequency of P335del in China is 0.6%, according to the NyuWa Chinese Population Variant Database, which suggests that ∼16.4 million individuals in China are heterozygous for this variant. Importantly, the allele frequency of P335del in Southern Han Chinese is ∼2% (Zhang et al., 2018; Sun et al., 2021), implying that 1/2,500 individuals in this region are likely to be homozygous for this variant. The P335del *IFNAR1* variant has been detected in other Asian countries, including South Korea (MAF = 0.0132), Vietnam (MAF = 0.0179), and India (MAF = 0.0008) (GenomeAsia100k), but is absent or extremely rare elsewhere, suggesting a potentially higher frequency in various Southeast and Northeast Asian communities. We identified eight patients carrying the P335del *IFNAR1* variant (one homozygous and seven heterozygous), seven of whom were of Southeast Asian origin. The only homozygous patient (P9, Turkish) suffered from critical COVID-19 pneumonia at the age of 16 years. Three heterozygous patients (P11–13) presented with critical COVID-19 pneumonia at the ages of 23, 57, and 62 years, and one of the older patients (P12) died of COVID-19. Two other, younger heterozygous patients (P10 and P14) suffered SARS-CoV-2 infection at the ages of 30 and 32 years and had mild COVID-19. These data are consistent with our previous observations for patients with APS-1 (Bastard et al., 2021d) or inborn errors of the alternative NF-kB pathway (Le Voyer et al., 2023), age being a key determinant of hypoxemic COVID-19 pneumonia among patients with auto-Abs against type I IFNs. Likewise, older male patients with X-linked TLR7 deficiency were found to be at greater risk of hypoxemic COVID-19 than younger patients (Asano et al., 2021). This may reflect the decline in mucosal type I IFN levels with age (Loske et al., 2022; Woodall et al., 2024; Zanoni, 2024). Finally, two heterozygous patients (P15 and 16) suffered from JEV encephalitis at the ages of 5 and 3 years, respectively, providing a first explanation for this condition (Pommier et al., 2022). Our data, thus, suggest that the clinical penetrance of the P335del variant is incomplete and that the penetrance of severe viral infections may increase with age and depend on the virus. They also highlight potential genetic vulnerabilities to viral epidemics at an unprecedented scale in China, particularly among the Southern Han demographic group.

## Discussion

We report three novelties pertaining to human type I IFN immunity. We describe 10 *IFNAR1* alleles underlying an AD and a partial form of IFNAR1 deficiency that operates by negative dominance. Cellular responses to IFN-β were normal, contrasting with those to other type I IFNs, for the corresponding heterozygous genotypes, which caused a dominant form of IFNAR1 deficiency, attesting to the dissociation of responses to the different type I IFNs. Finally, we report that the AD form of IFNAR1 deficiency can be caused not only by rare alleles, but also, surprisingly, by an *IFNAR1* allele (P335del) that is common in the populous regions of Eastern Asia (Zhang et al., 2018). These findings apply not only to cell-intrinsic immunity in vitro, as analyzed with heterozygous cell lines in the laboratory, but also to host defense in vivo, as analyzed in heterozygous patients infected with various viruses in natural conditions. Individuals with AD IFNAR1 deficiency are vulnerable to various viral diseases, including critical COVID-19 pneumonia and JEV encephalitis. Our findings for patients with partial IFNAR1 deficiencies are consistent with the defective cellular responses to type I IFNs observed in patients with auto-Abs neutralizing IFN-α and/or -ω but not -β, which are determined by the specificities, levels, and affinities of these antibodies (Bastard et al., 2020, 2021a, 2021c; Manry et al., 2022; Zhang et al., 2022c; Gervais et al., 2023). Partial IFNAR1 deficiency should therefore be considered in patients with unexplained severe viral illnesses, such as LAV disease, critical COVID-19 pneumonia, HSE, and JEV encephalitis, and in those with other unexplained severe viral infections, including critical influenza and MERS pneumonia, or with WNV encephalitis and TBE, which are seen in patients with auto-Abs against type I IFNs.

There are, thus, recessive and dominant forms of IFNAR1 deficiency at the cellular level. Surprisingly, we found that dominance operates via negative dominance rather than haploinsufficiency. Moreover, this situation is different from that for other AD inborn errors of immunity operating by negative dominance, such as AD deficiencies of IFNGR1 and IL6ST, for which negative dominance results from an accumulation at the cell surface of the LOF protein due to a deletion of the cytoplasmic recycling motif (Jouanguy et al., 1999; Béziat et al., 2020). The mechanism of negative dominance remains unclear for these IFNAR1 variants. We hypothesize that IFNAR1–IFNAR2–IFN complexes may form a higher-order, homomultimeric structure. Furthermore, the IFNAR1 defect is partial, as opposed to the complete deficiency observed with other AD defects of cytokine receptors (Bastard et al., 2021c, 2022a; Hernandez et al., 2019; Abolhassani et al., 2022). We also found that cellular responses to IFN-β were normal, whereas responses to other type I IFNs were impaired by these variants. The mechanism of dissociation is unknown, but its consequences are consistent with the finding that neutralizing auto-Abs against IFN-α and/or IFN-ω confer a higher risk of life-threatening viral illnesses, whereas this is rarely the case for neutralizing auto-Abs against IFN-β (Bastard et al., 2020, 2021a, 2021c; Manry et al., 2022; Zhang et al., 2022c). This difference in the responses to IFN-β and other type I IFNs is probably due to the specific structural and functional properties of each *IFNAR1* variant impairing the affinity of the receptor for all type I IFNs, accounting for the residual activity of IFN-β, which is the type I IFN with the highest affinity for its receptor (Randall and Goodbourn, 2008; Wittling et al., 2021).

Indeed, early studies described anti-human IFNAR1 antibodies that significantly neutralized signaling by the various IFN-α subtypes but not signaling by IFN-β (Lu et al., 1998). Consistent with this finding, variants such as N44del and V96F were expressed at normal levels on the cell surface and responded to IFN-β but not to IFN-α or IFN-ω, suggesting differential effects on affinity for different IFNs. V96 on IFNAR1 SD1 is located adjacent to the ligand-binding pocket in the IFN-α2-YNS-IFNAR1-IFNAR2 ternary complex (Fig. 1 B) and is not known to interact directly with IFNs, but the adjacent residues, N95 and Y97, are important for IFN-α2-YNS, IFN-ω, and IFN-β activity (Thomas et al., 2011). Conversely, variants such as W73C and P334L are expressed at lower levels on the cell surface, and higher plasmid transfection rates resulted in higher rates of response to IFN-α or IFN-ω, indicating potential defects related to surface expression or receptor transport. It is also possible that these effects result from multiple mechanisms, including impacts on IFNAR1 conformation and stability, especially for mutations at sites that do not directly interact with IFNs. Importantly, the C79 residue on IFNAR1 SD1 is involved in a conserved disulfide bond with C87 that probably stabilizes IFNAR1 SD1, the integrity of which is important for the propagation of conformational changes enabling efficient IFN signaling (Strunk et al., 2008). Likewise, changes to highly conserved proline residues in the P130H, P334L, and P335del variants, all of which are located within interdomain hinge regions of IFNAR1, probably alter the flexibility of the IFNAR1 protein, thereby modulating its activity (Li et al., 2017). Finally, although the residue altered in the A264T variant of IFNAR1 SD3 is not directly involved in the binding of IFN, the adjacent H263 residue is directly involved in binding both IFNα2-YNS and IFN-ω, whereas F265 is vital for the activities of IFN-α2-YNS, IFN-ω, and IFN-β (Thomas et al., 2011). Overall, the higher affinity of IFN-β than of the other type I IFNs for the type I IFN receptor probably accounts for or at least contributes to this phenomenon (Randall and Goodbourn, 2008; Wittling et al., 2021).

The spectrum of viral illnesses observed in patients with partial IFNAR1 deficiency appears to be narrow, primarily involving susceptibility to respiratory and cerebral viruses, notably SARS-CoV-2 and LAV. The penetrance of severe LAV and naturally acquired viral diseases seems to be incomplete, as previously seen in patients with AR complete IFNAR1 or IFNAR2 deficiency, whereas penetrance is higher for infections with certain viruses, such as SARS-CoV-2 than for HSV-1 (Hernandez et al., 2019; Bastard et al., 2021b, 2021c, 2022a; Zhang et al., 2020, 2022b; Duncan et al., 2015, 2022; Passarelli et al., 2020). The mechanism underlying incomplete penetrance remains unclear, but it may be influenced by factors such as viral strain variability and inoculum, the capacity of the virus to induce IFN-β production and signaling, and the ability of residual IFN-β and type III IFN activity to protect against severe viral infections. Clinically, patients with a normal response to IFN-β but impaired responses to IFN-α and -ω would be expected to resemble both IRF7^−/−^ patients (Ciancanelli et al., 2015; Zhang et al., 2020; Campbell et al., 2022) and patients with auto-Abs neutralizing IFN-α and -ω but not -β (Bastard et al., 2020, 2021a, 2021c; Manry et al., 2022; Zhang et al., 2022c; Gervais et al., 2023). This seems to be the case, although IRF7 deficiency appears to preferentially underlie respiratory diseases (Ciancanelli et al., 2015; Zhang et al., 2020; Campbell et al., 2022). It is also probable that the penetrance of monoallelic dominant-negative *IFNAR1* variants is lower than that of biallelic genotypes. Both heterozygosity and homozygosity for IFNAR1 variants should be considered in patients presenting with severe viral illnesses, such as LAV disease, critical COVID-19 pneumonia, JEV encephalitis, and HSE. Viruses that can infect the brain and the lungs seem to pose a particular threat in patients with AD IFNAR1 deficiency, as previously documented in patients with related inborn errors of, or auto-Abs against type I IFNs.

Remarkably, 9 of the 10 dominant-negative *IFNAR1* variants were rare, but one was found to be common in Southern China (P335del). We estimated that about 16.8 million Chinese are heterozygous for this variant. This variant was also observed in South Korea, Vietnam, and India (GenomeAsia100k), suggesting that the frequency of P335del may be higher in other specific communities in Southeast and Northeast Asia. It is, therefore, crucial to determine heterozygosity and homozygosity rates, penetrance, and clinical presentations in populations living in China and its neighbors. Our study is consistent with the recent description of highly penetrant but common alleles underlying other infectious or autoimmune phenotypes. For example, we found P1104A TYK2 to be common in Europeans and its homozygosity to underlie 1% of European cases of TB (Kerner et al., 2019). We also found two PTCRA alleles common in Africa and South Asia, homozygosity for which underlies autoimmune manifestations (Materna et al., 2024). LOF variants of *IFNAR1* and *IFNAR2* are common in Western Polynesia and the Arctic, where they underlie viral disease in homozygotes (Bastard et al., 2022a; Duncan et al., 2022). Population studies of the *IFNAR1* P335del variant are warranted, particularly in and around China. Expanding patient-based studies to include a larger number of kindreds and conducting population-based studies will improve our understanding of the frequency and clinical characteristics of individuals with AD IFNAR1 deficiency due to heterozygosity for P335del. This will include determining the penetrance of each viral illness, potentially making it possible to develop preventive strategies for use in patients with the corresponding genotype.

## Materials and methods

### Study and ethics approval

Informed consent was obtained in each country of follow-up, in accordance with local regulations and the requirements for institutional review board (IRB) approval for Rockefeller University (protocol no. JCA-0700) and the Institut National de la Santé et de la Recherche Médicale (INSERM) (RCB ID 2010-A00634-35). Experiments were conducted in the United States and France, in accordance with local regulations and with the approval of the IRB of Rockefeller University and INSERM, respectively. Samples were obtained from the probands, parents, and relatives with written informed consent.

### Patients

P1 (W73C/W73C) is a 38-year-old man living in Turkey who survived critical COVID-19 pneumonia (Zhang et al., 2020). He was hospitalized 10 days after the onset of symptoms, including fever and cough, in March 2020. Chest X-ray showed ground-glass infiltrates and consolidation in the lungs. P1 received high-flow oxygen therapy, hydroxychloroquine, azithromycin, and favipravir. He reported having frequent respiratory infections (including influenza) during the winter. He received the MMR vaccine without complications.

P2 (C79R/+) is a 4-year-old boy living in Mexico who had mild COVID-19 infection. He is also homozygous for a splicing-site mutation of *IL12RB1* that is LOF and underlies MSMD. He was infected with SARS-CoV-2 in January 2021.

P3 (C79Y/+) was a 63-year-old man originally from Ivory Coast who was living in France during the pandemic and died from critical COVID-19 pneumonia. He was hospitalized in October 2020 and subsequently developed ARDS, necessitating his transfer to the ICU and treatment with high-flow oxygen therapy. P3 was treated with dexamethasone, methylprednisolone pulses, and cyclophosphamide. He was diagnosed with interstitial lung disease, anti-synthetase syndrome with anti-PL7 auto-antibodies 40 days after the initial positive PCR test for SARS-CoV-2. He died of respiratory failure 20 days later. P3 had diabetes mellitus and hypertension before contracting COVID-19.

P4 (M155I/+) was an 84-year-old man living in Lebanon who died from critical COVID-19 pneumonia. He was hospitalized in December 2020, and chest X-ray showed bilateral ground-glass opacities in the perihilar and basal zones and regional consolidation. P4 was intubated for mechanical ventilation and treated with remdesivir. He subsequently developed acute kidney injury requiring dialysis. He died of renal failure 15 days after admission. He suffered from eczema, diabetes mellitus, and hypertension before the infection and had a family history of lung cancer.

P5 (Y215C/Y215C) is a 5-year-old boy living in Spain who survived HSE at the age of 2 years (Armangué et al., 2023). He developed typical post-HSE choreoathetosis, presenting with severe dysphagia, hypotonia, a decreased level of consciousness, seizures, and continuous choreoathetosis of all four limbs 26 days after the onset of HSE. He was treated with aggressive immunotherapy, including steroids, IVIG, plasma exchange, and rituximab, and displayed slow but progressive improvement. By 2.5 years post-HSE onset, at the age of 4 years, he had residual speech problems (limited expressive language) but demonstrated good comprehension, motor skills, and sociability. He remained on antiepileptic medication despite being free from clinical seizures.

P6 (A264T/+) is a 9-year-old boy who was initially asymptomatic following exposure to his symptomatic sister with COVID-19 pneumonia in 2020. 3 wk later, he developed a skin rash and intestinal syndromes without pneumonia or encephalitis. He tested negative for SARS-CoV-2 by PCR, but was seropositive at the onset of symptoms, with a high CRP level (170 mg/L). He had frequent respiratory viral infections, including influenza. He was hospitalized once for tonsillitis and treated with antibiotics. He received the MMR vaccine without complications.

P7 (S422R/S422R) was a 26-year-old man originally from Pakistan who was living in Saudi Arabia during the pandemic and died from critical COVID-19 pneumonia in June 2020 (Zhang et al., 2020). He presented with a febrile cough and dyspnea on arrival at the hospital, progressing to respiratory failure requiring intubation for mechanical ventilation. Chest X-ray showed bilateral consolidation and infiltrations, and bilateral pneumothorax. P7 was treated with hydrocortisone, remdesivir, meropenem, vancomycin, and hydroxychloroquine. His vaccination and family histories were unknown.

P8 (N44del/+) is a boy from Cambodia who presented with EV71 encephalitis at the age of 2 years (Pommier et al., 2022). He was admitted to the hospital 2 days after the onset of symptoms, with fever, a Glasgow coma scale (GCS) score of 13, and limb weakness. He recovered fully after 10 days.

P9 (P335del/P335del) is a 17-year-old man living in Turkey who survived severe COVID-19 pneumonia. He was born to second-degree consanguineous parents. He was hospitalized with bilateral diffuse infiltration requiring oxygen therapy. He was obese and was vaccinated with the MMR vaccine without complications.

P10 (P335del/+) is a 30-year-old woman who had asymptomatic SARS-CoV-2 infection in May 2020. Chest X-ray showed no signs of pneumonia. P10 was vaccinated with two doses of mRNA vaccine in 2021 and had mild COVID-19 with a cough, congestion, sore throat, and fever in May 2022.

P11 (P335del/+) is a 23-year-old woman of Chinese origin living in Italy, who survived critical COVID-19 pneumonia (Zhang et al., 2020). She was hospitalized in September 2020 with COVID-19 pneumonia requiring oxygen therapy (CPAP). She was vaccinated with the MMR vaccine without complications.

P12 (P335del/+) was a 57-year-old woman of Chinese origin living in the UAE, who died from COVID-19 pneumonia complicated by septic shock. The patient was hospitalized in May 2020. Chest X-ray showed bilateral infiltration and P12 was admitted to the ICU and intubated for mechanical ventilation. She developed lung fibrosis and it was difficult to wean her off of mechanical ventilation. In September 2020, she developed fever, difficulty breathing, and septic shock, leading to her death 3 days later. Her vaccination history was unknown.

P13 (P335del/+) is a 62-year-old woman from Thailand who had critical COVID-19 pneumonia. Her vaccination history was unknown.

P14 (P335del/+) is a 32-year-old man living in Hong Kong who had a mild SARS-CoV-2 infection in July 2020. He had mild symptoms, including sore throat and anosmia for 2 mo. Chest X-ray showed no signs of pneumonia. His vaccination history was unknown.

P15 (P335del/+) was a 5-year-old boy from Cambodia who presented with JEV encephalitis (Pommier et al., 2022). He was admitted to the hospital 4 days after the onset of symptoms, which included fever, a GCS score of 13, focal seizure, hypertension, and a lesion of the thalamus on MRI. P15 deteriorated rapidly and died within 4 days of hospitalization.

P16 (P335del/+) is a 3-year-old boy from Cambodia who presented with JEV encephalitis (Pommier et al., 2022). He was admitted to the hospital 4 days after the onset of symptoms, which included fever, altered mental state, a GCS score of 9, generalized seizure and bilateral limb weakness. MRI revealed a diffuse edema. P16 fully recovered after 2 mo.

P17 (N29fs/N29fs; c.86delA) is a 2.5-year-old girl living in India who had asymptomatic SARS-CoV-2 infection in July 2021. She was diagnosed with Kawasaki disease 10 days after receiving a first dose of MMRV vaccine at the age of 1 year. She recovered after treatment with IVIG and antibiotics. She was treated with antibiotics for a salmonella infection at the age of 1.5 years and for a UTI at the age of 2 years. She had an elder brother who was hospitalized and died from an HLH-like disease at the age of 2 years. His genotype is unknown.

P18 (V225fs/V225fs) is a 1-year-old boy living in Iran who survived disseminated vaccine-strain measles infection after MMR vaccination (Hernandez et al., 2019). He was born to consanguineous parents, with a younger sibling who died 4 wk after a first MMR vaccination.

P19 (V225fs/W261X) is a 14-year-old girl living in Brazil who survived viscerotropic disease caused by the YF vaccine at the age of 12 years (Hernandez et al., 2019). She was vaccinated with the MMR vaccine at the ages of 12 and 16 mo without complications.

P20–25 (E386X/E386X) are six children from four unrelated kindreds of Western Polynesian ancestry, aged from 12 mo to 7 years, who suffered from disseminated vaccine-strain measles and HLH-like disease after MMR/V vaccination (Bastard et al., 2022a). One of the patients also suffered from critical RSV pneumonia requiring ECMO.

P26 (E386X/E386X) is a 13-year-old girl of Western Polynesian ancestry who suffered *Hemophilus influenzae* type B (Hib) bacteremia and meningitis at the age of 10 mo, critical RSV pneumonia at the age of 12 mo, and ARDS due to an unidentified pathogen at the age of 7 years (Bastard et al., 2022a). She was vaccinated with MMR/V at the age of 4 years without complications.

P27 (T389fs/T389fs; c.1158_1159insA) is a 1-year-old boy from Israel who suffered from HLH-like disease after MMR/V vaccination. He is the fourth child born to consanguineous parents (cousins on both sides). He suffered a prolonged fever and rash following MMR/V vaccination. His sister died at the age of 4 years following EBV-related HLH-like disease, but her genotype is unknown.

P28 and P29 (Y481insIHCGICFPV*/Y481insIHCGICFPV*) are two children from the same family of Arab ancestry living in Palestinian territory (Bastard et al., 2021b). They were born to consanguineous parents, themselves the products of consanguineous unions. P28 was hospitalized for prolonged fever at the age of 13 mo and for aseptic meningitis at the age of 16 mo. P28 died from HSE at the age of 19 mo. P29 is now 17 years old and is homozygous for the same mutation as his brother. P28 suffered from two episodes of aseptic meningitis at the ages of 6 and 10 mo, parotitis at the age of 14 years followed by bilateral hearing loss, strongly suspected to be due to the mumps virus, as suggested retrospectively by his high level of anti-mumps IgG. Another of P29’s siblings died following MMR vaccination at the age of 12 mo. His genotype was unknown.

### Cells

Primary fibroblasts, SV40-immortalized dermal fibroblasts, and HEK293T cells were cultured and maintained in Dulbecco’s modified Eagle medium (DMEM, Thermo Fisher Scientific) supplemented with 10% fetal bovine serum (FBS, Thermo Fisher Scientific).

### Plasmids

The IFNAR1 cDNA was inserted into the pGEMT cloning vector (Promega). IFNAR1 constructs were then subcloned into pMET7 for overexpression studies. Site-directed mutagenesis was performed to introduce the specific mutations, as indicated. All constructs were resequenced to confirm that the intended mutations were correctly introduced and that no other mutations were unintentionally generated during the cloning process.

### Luciferase reporter assay

IFNAR1^−/−^ HEK293T cells were transfected with a plasmid containing the firefly luciferase gene under the control of the human ISRE promoter in the pGL4.45 backbone, a plasmid constitutively expressing *Renilla* luciferase for normalization (pRL-TK), and plasmids encoding the various IFNAR1 variants. Cells were transfected in the presence of the X-tremeGene9 transfection reagent (6365779001; Sigma-Aldrich) for 24 h. Cells were either left unstimulated or were stimulated with various type I IFNs (Human IFN-α Sampler Set [11002; PBL], IFN-ω glycosylated [TP721113; OriGene] or not glycosylated [300-02; Peprotech], and IFN-β glycosylated [11415; PBL] or not glycosylated [11420; PBL]) for 16 h at 37°C. Finally, cells were lysed for 20 min at room temperature, and luciferase levels were measured with the Dual-Luciferase Reporter assay system (E1980; Promega) according to the manufacturer’s protocol. Luminescence intensity was measured with a SpectraMax iD3 microplate reader (Molecular Devices). Firefly luciferase activity values were normalized against *Renilla* luciferase activity values. These values were then normalized relative to the WT signal. IFNs were titrated against WT IFNAR1 before the testing of the variants. The assay was set up such that luciferase reporter induction was in the linear range for each IFN subtype and the concentration of each IFN used resulted in luciferase signals of similar intensity (IFN-α at 1,000 U/ml, IFN-ω at 1 ng/ml, and IFN-β at 100 U/ml). Variants were classified as LOF or hypomorphic if their luciferase activity was at least two standard deviations below the mean, corresponding to <50% of wild-type activity.

### Western blotting

Cells were lysed in NP-40 lysis buffer (280 mM NaCl, 50 mM Tris, pH 8, 0.2 mM EDTA, 2 mM EGTA, 10% glycerol, and 0.5% NP-40) supplemented with PhosSTOP Phosphatase Inhibitor (4906845001; Roche), and cOmplete Protease Inhibitor Cocktail (11697498001; Roche). The protein lysate was subjected to SDS-PAGE, and the resulting bands were transferred to a nitrocellulose membrane. Nonspecific binding was blocked by incubation with 5% nonfat milk powder and the membrane was then incubated overnight at 4°C with a primary antibody directed against IFNAR1 (ab124764, 1:1,000 dilution; Abcam) and then for 1 h at room temperature with a secondary anti-rabbit HRP-conjugated antibody (NA934V, 1:10,000 dilution; Cytiva). For protein deglycosylation, PNGase F (P0704S; NEB) was used according to the manufacturer’s instructions. For reprobing, blots were stripped by incubation for 10 min at room temperature with Restore Western blot Stripping Buffer (21059; Thermo Fisher Scientific). They were then incubated with anti-GAPDH-HRP antibody (Sc-47724, 1:5,000 dilution; Santa Cruz) for 1 h at room temperature. Membranes were washed with TBS-Tween, developed with the Pierce ECL western blotting Substrate (32106; Thermo Fisher Scientific), and the resulting signal was detected with an Amersham Imager 600 (GE Healthcare Life Sciences).

### Flow cytometry

Cultured SV40-fibroblasts were centrifuged and the resulting pellet was resuspended in FACS buffer (PBS with 2% FBS). The cells were then stained by incubation with fluorescently labeled antibodies for 30–45 min at 4°C (mouse anti-IFNAR1: AA3 mAb [a gift from L. Runkel, Biogen, Inc.]; IFNAR2: Miltenyi Biotec, 130-128-948; HLA-I PE: R&D Systems, FAB7098P). For IFNAR1 staining, cells were washed once with PBS and incubated with a goat anti-mouse secondary antibody, AF488 (A-11001; Thermo Fisher Scientific), for 30 min. For pSTAT1, cells were starved overnight in DMEM and 1% FBS. They were then stimulated directly with IFNs for 15 min at 37°C, fixed by incubation in 4% formaldehyde for 10 min at 37°C (557870; BD Phosflow Fix Buffer I), and permeabilized by incubation in cold Phosflow Perm Buffer III (558050; BD Biosciences) for at least 30 min. The cells were then subjected to surface staining and labeling for pSTAT1-AF657 (562070; BD Bioscience) by incubation with the appropriate antibodies for 45 min at 4°C. They were washed twice with PBS and analyzed by flow cytometry. Data were acquired on a LSRII (BD Biosciences) flow cytometer, and the results were analyzed with FlowJo software (TreeStar).

### RT-qPCR

RNA was isolated from fibroblasts with the RNeasy Plus Mini Kit (74134; QIAGEN) and converted into cDNA by reverse transcription with the SuperScript III First-Strand Synthesis System (18080051; Thermo Fisher Scientific), according to the manufacturer’s instructions. RT-qPCR was performed with the TaqMan Universal PCR Master Mix (4304437; Thermo Fisher Scientific) and Applied Biosystems Taqman assays for IFNAR1 (4331182; Thermo Fisher Scientific) and the β-glucuronidase (GUS; 4448489; Thermo Fisher Scientific) housekeeping gene. All reactions were normalized against the GUS housekeeping gene.

### SARS-CoV-2 infection

SARS-CoV-2 infections were conducted as previously described (Rosain et al., 2023; Lee et al., 2023). In brief, the SARS-CoV-2 NYC isolate (GenBank OM345241) was obtained from a de-identified patient in July 2020. The viral isolate was amplified by 6-to 7-day passages in Caco-2 cells at 37°C. After each passage, the virus-containing supernatant was harvested, clarified by centrifugation (3,000×*g* for 10 min), and filtered through a 0.22-μm-mesh disposable vacuum filter system. The passage three stock, used in this study, had a titer of 3.4 × 10^6^ PFU/ml, as determined on Vero E6 cells with a 1% methylcellulose overlay. SV40-fibroblasts stably transduced with ACE2 were used to seed 96-well plates at a density of 5,000 cells per well in the presence or absence of the indicated doses of IFNs and/or anti-IFN-β neutralizing antibody (mabg2-hifnb-3; InvivoGen). The cells were infected with SARS-CoV-2 20 h later by adding 0.1 μl of viral inoculum to the medium (final volume 110 μl) and centrifuging the cells for 5 min at 500×*g* and room temperature. Infections were performed in triplicate (separate wells). The cells were fixed, 24–48 h after infection, by adding neutral buffered formalin at a final concentration of 10%. They were stained for SARS-CoV-2 with an antibody directed against the N protein at a dilution of 1:3,000 (GTX135357; GeneTex) and then with an Alexa Fluor 647-conjugated secondary antibody (A-21245; Invitrogen) and 1 μg/ml Hoechst 33342 (H3570; Invitrogen). Plates were imaged with an ImageXpress micro XL and analyzed with MetaXpress (Molecular Devices).

### Online supplemental material

Fig. S1 shows the population genetics of the *IFNAR1* variants present in the HGID and gnomAD v4.0.0 databases. It also shows a western blot for *IFNAR1* variants after the treatment with PNGase. Fig. S2 shows the functional characterization of *IFNAR1* variants in terms of the response to type I IFNs. Fig. S3 shows the function of IFNAR1 variants in the patients’ fibroblasts. Fig. S4 shows SARS-CoV-2 infection of IFNAR1-deficient patient cells in vitro. Fig. S5 depicts auto-Abs neutralizing type I IFNs in the patients with *IFNAR1* variants. Table S1 provides the binding affinities of type I IFNs to IFNAR1. Table S2 provides the summary of the expression and impact of the deleterious *IFNAR1* variants. Table S3 shows the variants identified in the known IEI-causing genes present in patients with deleterious *IFNAR1* variants.

## Supplementary Material

Table S1 provides the binding affinities of type I IFNs to IFNAR1.

Table S2 provides the summary of the expression and impact of the deleterious *IFNAR1* variants.

Table S3 shows the variants identified in the known IEI-causing genes present in patients with deleterious *IFNAR1* variants.

SourceData F3 is the source file for Fig. 3.

SourceData FS1 is the source file for Fig. S1.

## Data Availability

All data supporting the findings of this study are available within the main text and supplemental material and from the corresponding authors upon request.
