# Supplementary figures and images for: A common form of dominant human IFNAR1 deficiency impairs IFN-α and -ω but not IFN-β-dependent immunity
Source: J Exp Med. 2024 Dec 16;222(2):e20241413. doi: 10.1084/jem.20241413 (PMC11648951; doi:10.1084/jem.20241413)

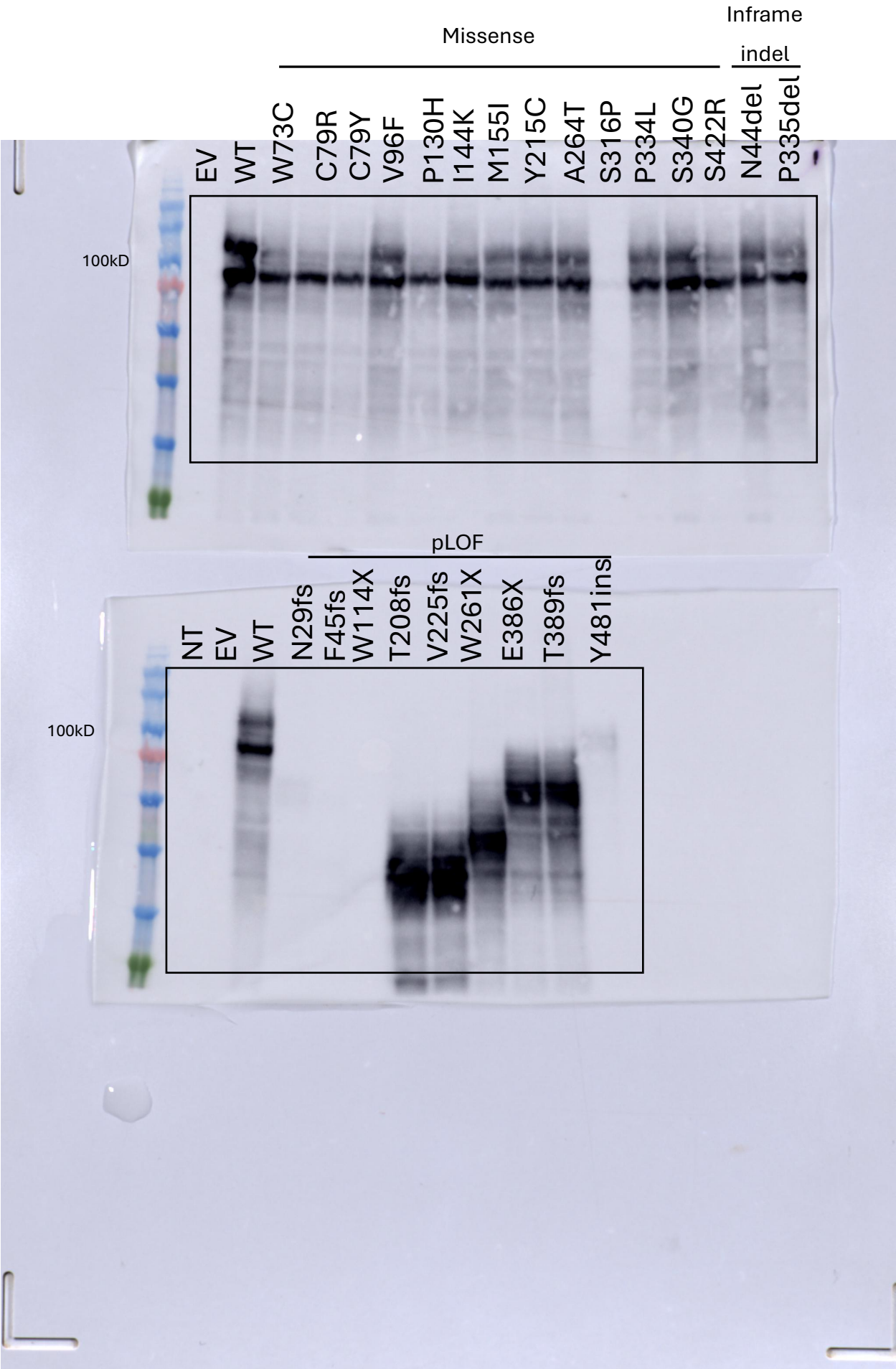

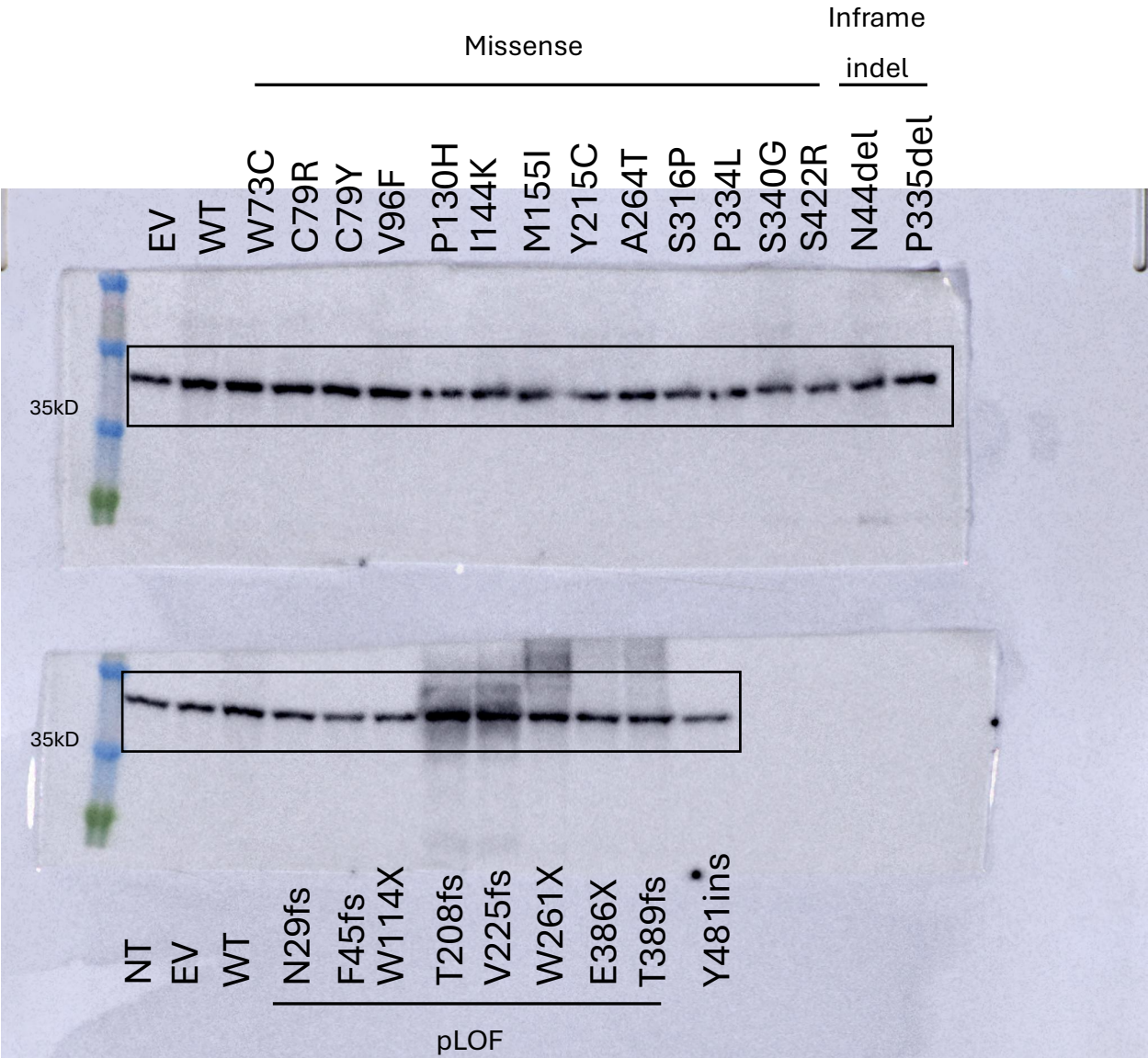

Supplement: SourceData F3 — is the source file for Fig. 3. [file jem_20241413_sourcedataf3.pdf]

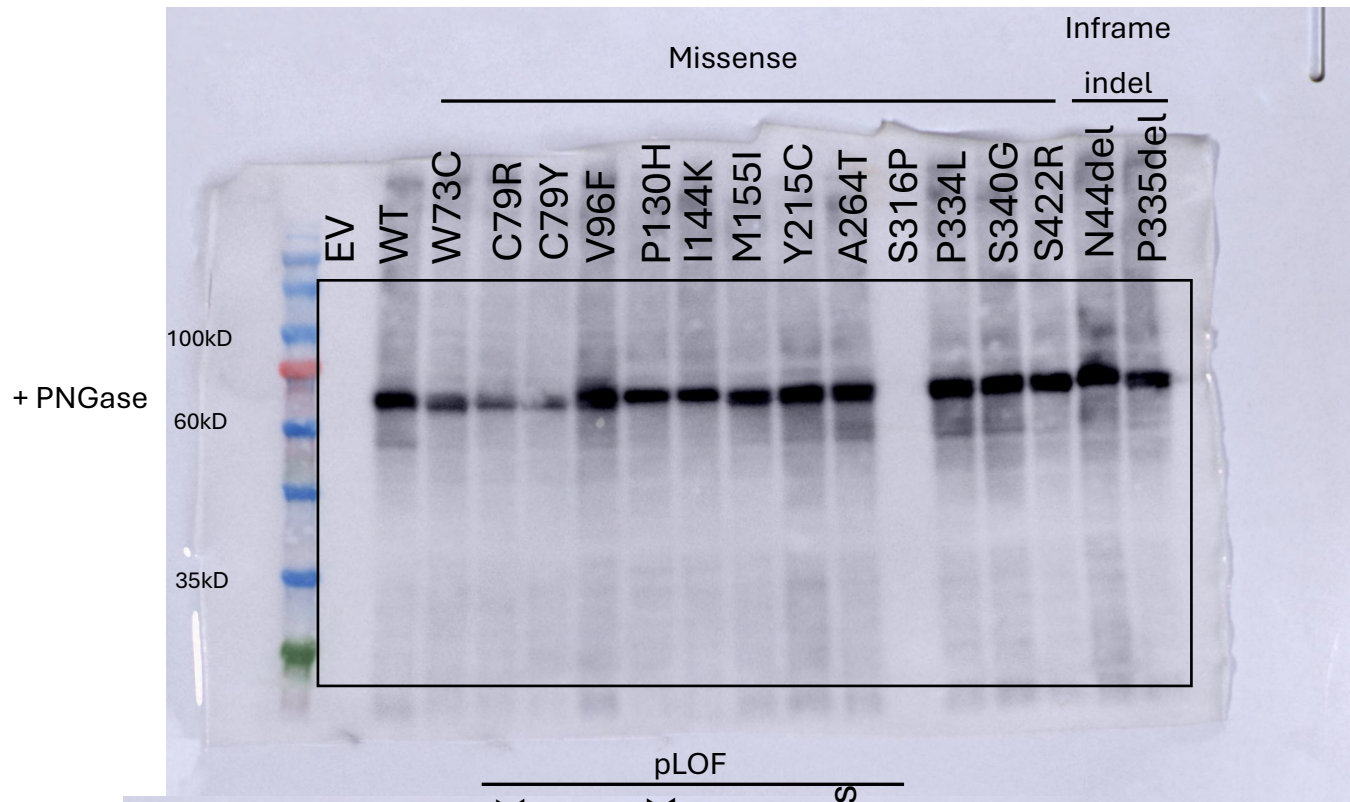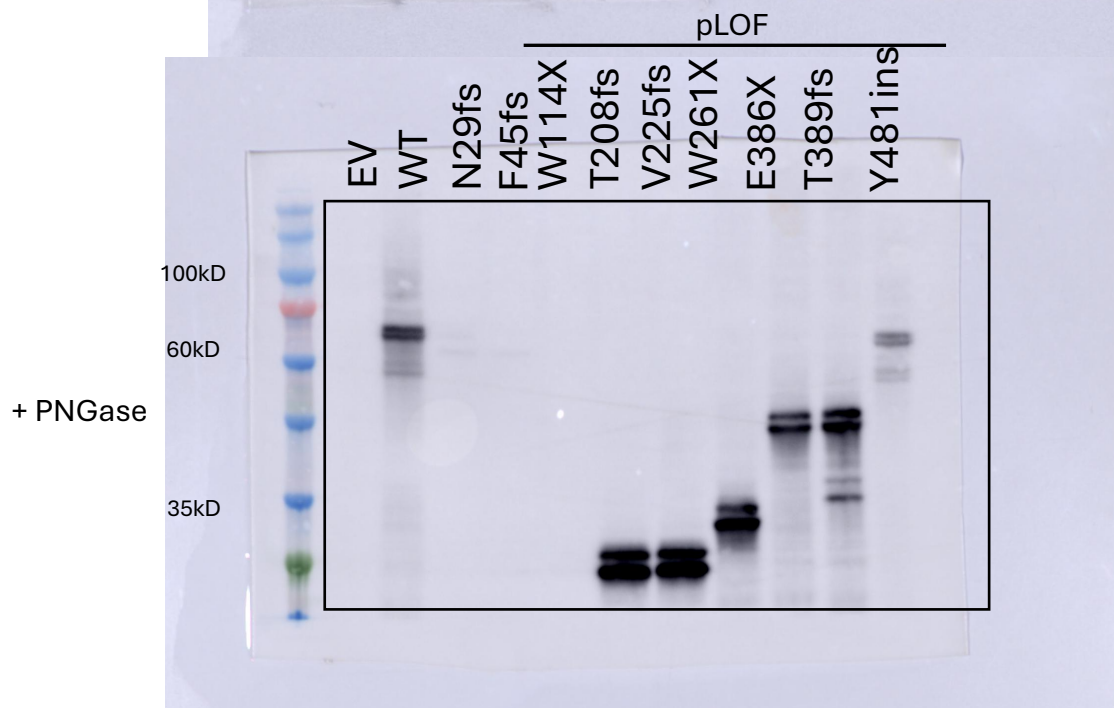

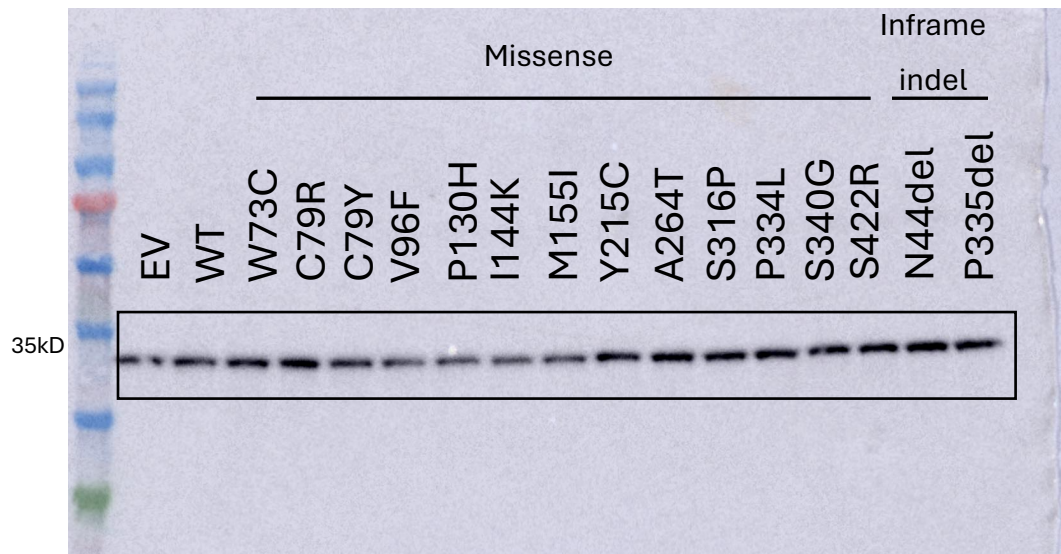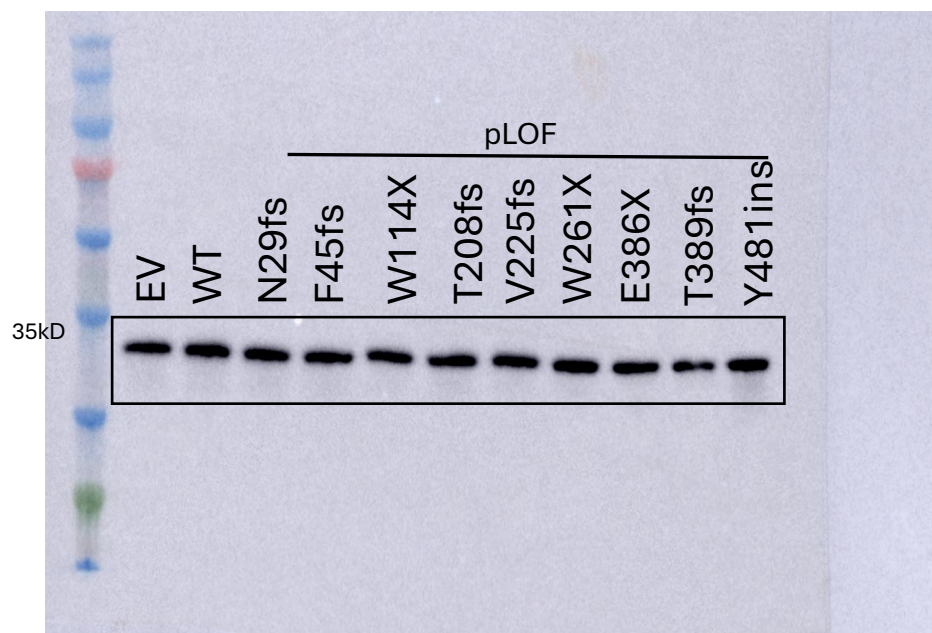

Supplement: SourceData FS1 — is the source file for Fig. S1. [file jem_20241413_sourcedatafs1.pdf]
